# Insights into the Etiology of Mammalian Neural Tube Closure Defects from Developmental, Genetic and Evolutionary Studies

**DOI:** 10.3390/jdb6030022

**Published:** 2018-08-21

**Authors:** Diana M. Juriloff, Muriel J. Harris

**Affiliations:** Department of Medical Genetics, University of British Columbia, 2350 Health Sciences Mall, Vancouver, BC V6T 1Z3, Canada; muriel.harris@ubc.ca

**Keywords:** neural tube, neural folds, anencephaly, exencephaly, spina bifida, craniorachischisis, folate, epigenetics, developmental genetics

## Abstract

The human neural tube defects (NTD), anencephaly, spina bifida and craniorachischisis, originate from a failure of the embryonic neural tube to close. Human NTD are relatively common and both complex and heterogeneous in genetic origin, but the genetic variants and developmental mechanisms are largely unknown. Here we review the numerous studies, mainly in mice, of normal neural tube closure, the mechanisms of failure caused by specific gene mutations, and the evolution of the vertebrate cranial neural tube and its genetic processes, seeking insights into the etiology of human NTD. We find evidence of many regions along the anterior–posterior axis each differing in some aspect of neural tube closure—morphology, cell behavior, specific genes required—and conclude that the etiology of NTD is likely to be partly specific to the anterior–posterior location of the defect and also genetically heterogeneous. We revisit the hypotheses explaining the excess of females among cranial NTD cases in mice and humans and new developments in understanding the role of the folate pathway in NTD. Finally, we demonstrate that evidence from mouse mutants strongly supports the search for digenic or oligogenic etiology in human NTD of all types.

## 1. Introduction

The mechanisms of closure of the neural tube and the development of neural tube closure defects (NTD) are genetically complex and diverse. With an incidence of one to ten per thousand births, NTD are among the most common severe birth defects [1]. Defects in cranial and spinal neural tube closure (anencephaly and spina bifida) seem to occur at almost equal frequencies in human embryos [1,2]. In the almost 300 mouse mutants with NTD, cranial defects strongly predominate [3,4]. However, much of the molecular genetic understanding of mammalian neural tube development is based on studies of the spinal region in mouse embryos. Many aspects have been comprehensively reviewed previously [4,5,6,7,8,9]. This review gives an overview of the developmental process and the genetic underpinnings of neural tube closure in mammals, and then focuses on some aspects that have potential to bring new insight.

The formation of a neural tube by the mechanism of rolling up a neural plate into a tube evolved before the evolutionary emergence of vertebrates, and some mammalian molecular mechanisms likely reflect this ancient origin. We briefly consider the process of neural tube formation in the animal usually used as a proxy for the ancestral chordate, Amphioxus, and in Ascidian embryos, representative of another ancient pre-vertebrate lineage, the Tunicates. There are extensive differences in the physical process of neural tube closure among modern species and even among mammals; we shall briefly also consider these to point to the need for assessment of these differences before using observations from model animals to predict the etiologies of human NTD. 

We give considerable emphasis to the anterior–posterior regional differences in the mechanisms of neural tube closure at the morphological and molecular levels, underlining the concept that defects of closure of the neural tube are mechanistically heterogeneous with distinct or partially overlapping etiologies. This heterogeneity predicts genetic differences in the etiologies of human NTD located at different sites along the anterior–posterior axis, as well as genetic heterogeneity at any given site. We focus on cranial neural tube development, its genetic mechanisms of failure to close, and the evolutionary New Head hypothesis that predicts consequential differences between the cranial and spinal regions in the mechanisms of neural tube closure. These differences should inform genetic studies of human NTD. 

We consider recent advances in understanding of the epigenetic mechanism by which the sex chromosome complement of mammalian embryos influences risk of cranial NTD, seeking new insight into the neural tube closure process. Similarly, we focus on the phenomenon of the preventative effect of maternal folic acid supplementation on occurrence of NTD in humans and in mice and recent developments in understanding of folate mechanisms, seeking possible insights into the etiologies of NTD. 

That there is a genetic etiology for human NTD is clear, with 30× elevated risk in siblings of NTD cases, but it is complex. Typical recent studies are based on exon sequencing of candidate genes in cohorts of NTD cases, including a variety of locations and types of lesion. This approach for several planar cell polarity (PCP) genes has detected heterozygous rare putatively deleterious variants in a small number of cases [10].

Despite many genetic studies, the etiology is not well understood [11], and likely involves combinations of deleterious versions of genes in affected individuals, with the combinations differing between individuals [12]. We discuss examples of similar genetic complexity in mouse mutant models.

Failure to close the neural tube leads to three principal open neural tube defects: craniorachischisis, in which the entire spinal region and the hindbrain fail to close; spina bifida aperta, in which a limited region of the spinal neural tube fails to close, and anencephaly or exencephaly, in which the midbrain and sometimes also the hindbrain fail to close. There are other defects that arise subsequently from defects in the tissue of the closed neural tube or surrounding mesenchyme, such as spina bifida occulta (abnormal formation of the vertebral arches from mesenchyme) and encephalocele (extrusion of neural tissue through a defect in the formation of the roof of the closed neural tube). Semantically or clinically, they are often included in NTD, but they are not within the scope of the etiology of failure to form a closed neural tube [6,13].

## 2. The Basics of Neural Tube Formation

### 2.1. Components

**Figure 1:**
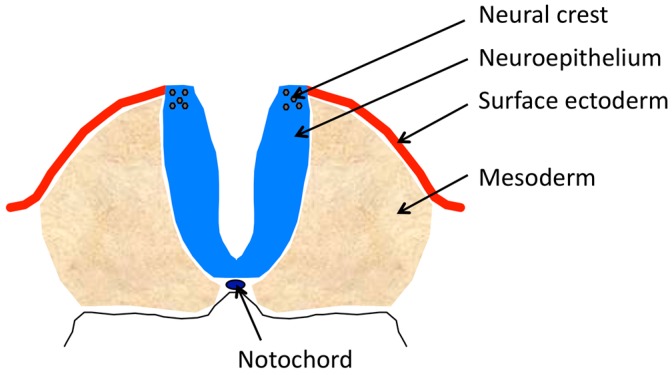
Diagram of a transverse section of upper spinal neural folds of an E8 mouse embryo before neural tube closure. Somites not shown.

The major physical components of the complex that will comprise the neural tube (Figure 1) include: the anterior to posterior midline neural plate, comprised of neuroectoderm (neuroepithelium) that develops on the dorsal aspect of the embryo, curls from its lateral edges toward the midline and fuses its formerly lateral edges in the midline; the notochord that underlies the neural plate and produces signaling molecules that affect dorsal–ventral cell identity in the incipient neural tube; the lateral ectoderm that initially abuts the lateral edges of the neural plate, covers the lateral surfaces of the elevating neural folds and after the bending of the folds to the midline, meets and fuses to form a contiguous surface ectoderm over the tube; the paraxial mesoderm lateral to, and supporting, the neuroepithelium of the elevating folds; and the neural crest that is induced in the neuroepithelium of the lateral tips of the spinal neural plate or in the dorsal tips of the elevated cranial neural folds. There are several mechanisms affecting these components that lead to failure of neural tube closure (Table 1).

### 2.2. Convergent Extension and Other Functions of the Planar Cell Polarity Pathway

Immediately preceding the initiation of neural tube formation, the vertebrate embryo undergoes convergent extension, which involves the lateral-to-medial convergence and anterior–posterior extension of axial tissues, including the neural plate. The non-canonical Wnt/PCP (planar cell polarity) pathway is crucial to this process. Cells change position in the tissue, intercalating between surrounding cells to shift toward the midline. A prerequisite is the directional orientation of cells relative to a morphogenic signal. The cells become asymmetrical in the positioning of various subcellular structures, including the core proteins of the PCP pathway [14,15]. Mutations in PCP genes interfere with the directionality of neuroepithelial cell movement in a variety of gene-specific ways [16]. The planar cell polarity (PCP) pathway is complex, comprising two main sets of interacting genes, the “core” pathway, and the Fat-Dachsous pathway, probably connected by *Prickle* [14]. The “core” pathway is highly conserved and is regulated by gradients of particular Wnt proteins, (e.g., *Wnt5a* and *Wnt11*). The “core” proteins include the transmembrane receptors *Vangl1/2*, *Celsr1/2/3*, and *Fzd* (Frizzled) and the cytosolic proteins *Dvl*, *Prickle*, Inversin (*Invs*) and *Scrib* [14]. The Fat/Dachsous pathway, acting upstream or in parallel with the PCP core pathway, includes the large membrane cadherins, *Fat* and *Dchs*, which bind to each other and are regulated by *Fjx* [5]. An additional assortment of genes serve various functions necessary to the PCP pathways; for example, *Ptk7*, *Sec24b*, *Intu* and *Fuz* [10]. 

In addition to its essential role in planar cell polarity required for convergent extension, the PCP pathway affects ciliogenesis, the arrangement of cytoskeletal actin and microtubules, the basal body and the centriole within cells [14,17,18]. Small Rho family-GTPases regulate the localization of actin polymerization. Microtubules are polarized, and their polarity is required for “correct axis establishment of PCP”, then PCP signaling is required for stability, correct localization and remodeling of microtubule networks. It is thought that PCP, actin and microtubules together determine the asymmetrical positioning of the centriole, ciliary basal bodies, cilia and the orientation of the mitotic spindle [14]. There is also interconnection between the convergent extension system and the neural plate bending system based on actomyosin. For example, RhoA and its effector ROCK are involved in both systems [19,20].

### 2.3. Bending and Hinge Points

**Figure 2:**
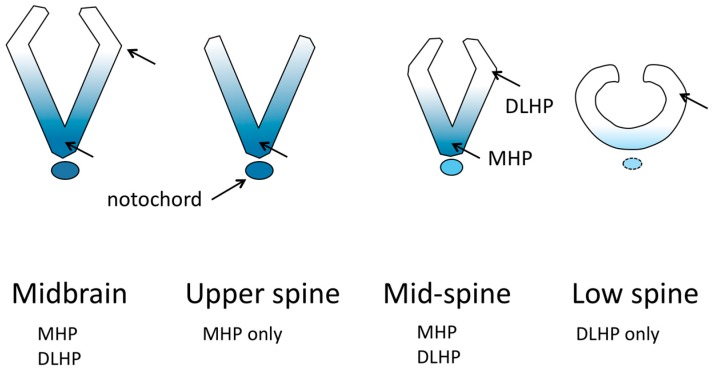
Conceptual views of the shape of neuroepithelium in transverse sections, at various anterior–posterior locations, of a mouse neural tube before closure, demonstrating differences in bending mechanisms. Shading represents the concentration of *Shh* signaling. DLHP, dorsolateral hinge point. MHP, medial hinge point. Modified from Figure 3 in [13].

Two areas of the neural plate undergo pronounced bending that strongly contributes to forming a tube; these are the medial hinge point (MHP) located at the midline base of the neural tube throughout most of its length, and the dorsolateral hinge point (DLHP) located in the dorsal third of each fold in some zones along the length of the neural tube [21] (Figure 2). In addition, the contraction of actomyosin found in the apices of all neuroepithelial cells has long been thought to contribute to bending the neural folds, and indeed, apical actomyosin is essential for cranial neural tube closure [19], but its specific role is unclear. In contrast, evidence indicates that apical actomyosin is not essential for spinal neural tube closure [19]. Instead, in the neuroepithelium of the spinal region, an orchestrated dynamic of actin turnover and actomyosin disassembly is essential for closure, and has been suggested to function in maintaining a necessary tension in the neuroepithelium (neither too floppy nor too stiff) between the MHP and DLHP sites [19]. The MHP and DLHP in the spinal region differ in their mechanisms that cause neuroepithelial bending. For the MHP to form in mouse embryos, signaling from the notochord is required [22]. At the MHP, bending is attributed to neuroepithelial cell shape. During the cell cycle, in S phase, nuclei move to the base of the cells, causing them to become wedge-shaped, with a narrow apex and wide base. Compared with the rest of the neuroepithelium, the MHP has an increased proportion of cells in S-phase, and also has cell-cycle dependent changes in apical and basolateral junctional proteins that contribute to cell shape [23,24]. 

The mouse spinal DLHPs form at the dorsoventral location where the neuroepithelium ceases to have basal contact with the paraxial mesoderm and begins basal contact with the surface ectoderm [23]; however, the significance of this relationship is unknown, given that the upper-spinal neural tube, which can form DLHP in the absence of *Shh* [22], does not have this mesoderm–ectoderm contact transition. Cell wedging related to cell cycle is not seen at the DLHP. Instead, there is cell proliferation and cell movement dorsally in the neuroepithelium, and evidence of a boundary at the DLHP between areas of different cell density. It has been proposed that the neural folds are caused to bend by the cell density difference [23]. There is an important relationship of the formation of the DLHP in the neuroepithelium with *Shh* signaling molecules from the ventral neural plate, *BMP* signaling from the surface ectoderm, and *Noggin* from the neuroepithelium [22]. The dorsal bends that appear to be DLHP in the midbrain neural folds have not been studied and it is possible that, despite appearing similar morphologically, they are driven by different cellular or molecular mechanisms. 

### 2.4. Cilia

Most cells have a primary cilium. In the neural tube, primary cilia can be seen extending from the neuroepithelium into the incipient lumen (see Figure 6 in [25]). Vital components of signaling pathways that are important to development of the neural tube are located in cilia, and if gene mutations cause the structure/function of the cilia to be defective, these signaling pathways are dysregulated [13,26]. The best understood is *Sonic hedgehog* (*Shh*) signaling. Other pathways, such as signaling through *Wnt*, Calcium, *Pdgfr*, *Notch*, and *Tgfb*, may also function via cilia [13,26,27]. Some of the PCP genes affect the position and angle of some types of cilia on cells [28], and some types of PCP genes (the “effectors”) have a role in ciliogenesis in vertebrates [13,29]. There are many components to the cilia, such as intraflagellar transport proteins and parts of the dynein motors, and when they are mutated, they lead to defects in the structure and function of cilia.

### 2.5. The Sonic Hedgehog Pathway Signaling in NTD

Several components of the *Sonic hedgehog* (*Shh*) signaling pathway, including positive and negative regulators, are present in the cilia [13]. Shh is a secreted glycoprotein whose binding with the transmembrane receptor Patched (Ptch1) in the cilia releases Smoothened (Smo) from repression and activates the *Shh* signaling pathway. The *Shh* signaling pathway affects the transcription of downstream target genes via the Gli transcription factors; there are many transcriptional targets and complex feedback loops [30]. An active *Shh* signaling pathway inhibits the processing of a full-length activator form of Gli3 (Gli3FL) into a repressor form (Gli3R) and the ratio of Gli3FL:Gli3R is a measure of functionality of the *Shh* signaling pathway [13,30].

During neural tube development, *Shh* is produced by the notochord and prechordal plate extending from the future forebrain area to the caudal area [22], and induces *Shh* production from the midline of the neural plate with a ventral to dorsal gradient in the developing neural tube [13]. While the notochord is required for formation of the MHP, and *Shh* signaling is required for development of the floor plate, it is not clear that *Shh* is required for formation of the MHP; the MHP is not always required for neural tube closure [6,22]. The formation of the DLHP is inhibited by *Shh* signaling [22]. Absence of *Shh* signaling in the dorsal neural folds permits a neuroepithelial response to *Noggin*, which suppresses *Bmp* signaling from the adjacent surface ectoderm, enabling formation of dorsolateral hinge points (DLHP) [31]. *Shh* is expressed in a rostral to caudal and temporal gradient in the developing neural tube from the upper spinal region to the caudal spinal region, being expressed in the caudal notochord and floor plate after neural tube closure [31]. In the cranial neural tube, no gradient has been reported and expression is seen from before closure onwards (e.g., Figure 4c in [32] and Figure 1a in [22]). The presence of DLHP in the cranial neural folds, particularly the midbrain, despite possibly robust *Shh* signaling in the ventral midline seems contradictory to the Shh concentration-dependent inhibition of DLHP in the upper spine. However, in many species, the cranial neural folds are clearly much larger than the spinal neural folds; this is particularly notable in mouse embryos. Therefore, the physical distance for diffusion of signaling molecules from the midline to the dorsal tips is greater than in the spine. We suggest that the large size of the cranial neural folds creates the necessary lack of signaling concentration in the cranial dorsal folds, enabling cranial DLHP formation. 

Loss of function of the *Shh* gene, or loss of function of components of the *Shh* signaling pathway such as *Smo*, do not cause NTD; the neural tube forms a midline bend and DLHP and closes. Typically, loss of function of the *Shh* signaling pathway causes a lack of development of the cranial midline tissues that results in holoprosencephaly and related facial defects [13,33,34]. In contrast, elevated activity of the *Shh* signaling pathway downstream of *Shh* due to the loss of negative regulators such as *Ptch1*, *Rab23*, *Tulp3* or *Sufu* [13] leads to elevated signaling in the dorsal neural folds, inhibiting development of the DLHP (e.g., *Tulp3* [35]) and causing NTD (exencephaly and sometimes caudal spina bifida) in the regions that normally have DLHP (Figure 2). 

Mutations in genes that affect the structure or function of cilia fall into two major types with respect to *Shh* signaling [13]. Some mutations that affect the structure/function of cilia, such as *Ttc21b* (intraflagellar transport), *Kif7* (kinesin motor protein) and *Arl13b* (cilial structure), seem to increase *Shh* signaling pathway activity based on expansion of ventral markers, and cause exencephaly, parallel to the other mutants with increased *Shh* signaling. Others, such as mutants for several intraflagellar transport (*Ift*) genes, dynein components (*Dync2h1*, *Dync2li1*), PCP effector genes (*Fuzzy*, *Inturned*), and *C2cd3* (which recruits *Ift* proteins for ciliogenesis) seem to reduce *Shh* signaling pathway activity, based on reduced ventral markers in the neural tube, but contrary to the mutations with loss of *Shh* signaling, they also cause exencephaly. Notably, it seems that cilia mutants that cause exencephaly have an increased Gli3FL:Gli3R ratio [13], but there are many other complex changes and regulatory loop alterations specific to each mutant, and possible involvement of the other cilia-based signaling pathways. Shh signaling also functions through a network of non-canonical, Gli-independent pathways, which could be involved [36]. The process of cranial neural fold formation and bending does not seem to have been examined in each mutant with exencephaly and it is unknown whether there is a shared mechanism, such as lack of DLHP formation, or a heterogeneity of mechanisms such as apoptosis in the neuroepithelium or deficient cell adhesion. 

### 2.6. Adhesion and Fusion

To complete neural tube closure, the neuroepithelium and surface ectoderm of the neural fold must make contact with their counterparts in the apposed fold and form de novo adhesions to create a continuous surface ectoderm covering a continuous neuroepithelium. The neuroepithelia and surface ectoderms do not necessarily make contact simultaneously, and the order varies along the anterior–posterior axis. Some details have emerged from recent studies. Filopodia and ruffles project from apposed surface ectoderms into the gap between the apposed folds and are required for successful closure, although their role is unknown [37,38,39]. Various membrane-bound *Eph* receptor tyrosine kinases and their membrane bound ligands, the *Ephrins*, which are generally involved in cell–cell attraction and repulsion interactions, are found in the neural folds, including the dorsal tips, just prior to fusion and are possibly involved in initiation of adhesions between apposed folds [38,40]. 

## 3. Regional Differences in Closure Mechanisms

Studies of mouse embryos have demonstrated that different regions along the anterior–posterior axis have different major mechanisms for bending the neuroepithelium to meet in the midline and also differ in fusion mechanisms (Table 2). These reflect several factors [5,6] including the early spinal role of planar cell polarity, the timing of anterior–posterior development of the notochord relative to closure, a gradient of *Shh* signaling along the anterior to posterior spine [22], presence of MHP and DLHP, differences in expression domains along the anterior–posterior axis of various genes important to closure, such as the *Grhl* gene family [41] and the *Ephrin*/*Eph* gene families [40,42,43], types of cell projections, cell types that initiate fusion, and factors in the molecular genetics of closure that are unique to the cranial region, such as the dynamics of the actomyosin cytoskeleton. Given the distinct regional differences in mechanism and genes involved in neurulation, studies to identify causative genetic variants in human NTD would be strengthened by studying pools of cases that share a similar location of lesion.

### 3.1. Planar Cell Polarity, MHP and DLHP

The upper spine region depends on planar cell polarity for cell movements and convergent extension that narrows the midline gap between the future neural folds, and on cell cycle dynamics in the neural plate midline that cause cell wedging to create a medial hinge point (MHP). The mid-spinal region also depends on the effects of convergent extension, and has the MHP, but additionally has DLHPs. The most caudal area of primary neurulation, the posterior neuropore (PNP), likely also depends on initial convergent extension to narrow the neural plate, but unlike the rest of the neural tube, the notochord is not present before closure [22]. The MHP does not form, but DLHPs bend the folds to the midline; there is also a greater dependence on actomyosin contraction for bending than in the more rostral spinal region [44]. Note: the PNP is sometimes used to refer to an extended region including part of the mid-lower spine to the most caudal end of primary neurulation; in this review we refer to only the most caudal part of this region as the PNP. 

Caudal to the PNP, neural folds do not form and a tube is hollowed out from the caudal mesenchyme in a process termed “secondary neurulation”. Defects in secondary neurulation per se would not cause closure defects, but there is evidence that mis-regulation of the development of the junction between the PNP and secondary neurulation may cause some lumbosacral open neural tube defects [45,46]. 

Overall, given the various overlapping regional differences in molecular genetic mechanisms and closure mechanisms from head to tail, it is likely that NTD in different anterior–posterior regions have region-specific causes that can be predicted. For example, in families with recurrence of NTDs, in about 70% of families, the second cases have the same defect as the first case, but in about 30% of families, one has spina bifida and one has anencephaly [47]. Given that failure of DLHP is a mechanism of NTD shared by anencephaly and lumbosacral spina bifida aperta, we suggest these latter families may be enriched for defects in genes that affect DLHP, which can inform the genetic approaches to unraveling some of the genetic complexity of human NTD. 

### 3.2. Closure Initiation Sites

**Figure 3:**
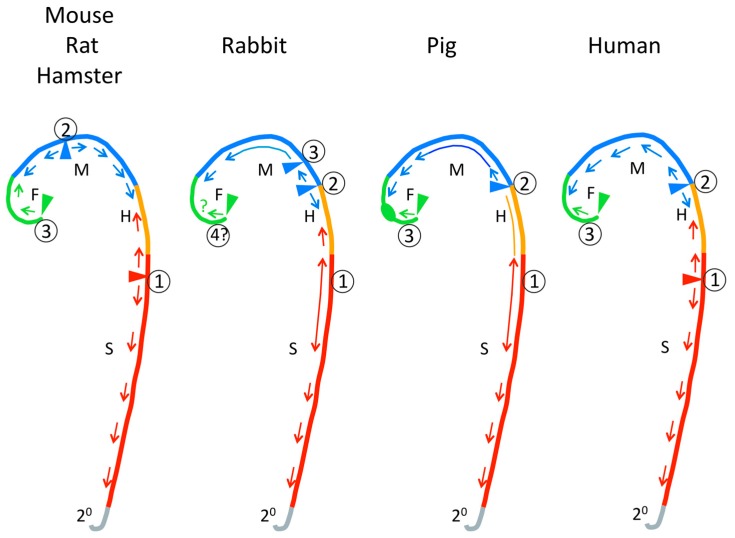
An interpretation of the patterns of neural tube closure in various mammalian species based on published images and studies. Colors denote future anterior–posterior fates of the neural tube. F, forebrain; M, midbrain; H, hindbrain; S, spine. Short arrows indicate direction of zipping. Long stems on arrows and lines lacking arrowheads denote areas that appose and then fuse simultaneously. Triangles and circled numbers indicate closure initiation sites. The forebrain oblong denotes a region that appears to close from all sides, rather than apposition or zipping. 2°, region of secondary neurulation.

At several specific locations along the anterior–posterior axis the neural folds achieve close apposition and initiate fusion. Closure then is thought to “zip” along the neural folds until it meets a closed region “zipped” from a different initiation site—an intermittent pattern rather than a single “zipper”. The mechanisms that determine the locations of closure initiation sites are not known. The locations of cranial closure initiation sites differ somewhat between normal mouse strains [48]; the possibility of similar variation within other species should be considered. The locations of cranial closure initiation sites and patterns of closure vary greatly between mammalian species as summarized in Figure 3 (mouse [49,50,51], rat [52,53], hamster [54,55], rabbit [56], pig [57], human [58,59,60,61,62]). The pattern of cranial closure initiation sites differs greatly between humans and mice, rats or hamsters, particularly with respect to the presence of an initiation site near the forebrain–midbrain junction that is not seen in human embryos (Figure 3). The closure process in rabbit and pig seem more similar to human. In rabbit, pig and human, instead of “zipping”, extended regions in the head and spinal region come into close apposition and then fuse simultaneously. Greater axial curvature seems to reduce the rate of closure along the anterior–posterior axis and between species [63]; flat areas tend to close simultaneously rather than “zip”. Otherwise, the location of the Closure 1 initiation site at the future neck (cervical) region and pattern of expansion of closure from it to the posterior neuropore seems generally similar across mammalian species. 

The relevance to NTD of species differences in locations of closure initiation sites is debatable. Generally, the locations of open cranial NTD and open spinal NTD in mice and humans appear to be the same, i.e., the same parts of the neural tube have failed to close. As we have said previously “… if the defect in both species is a failure of elevation itself, a species difference in the location of first contact of already elevated folds would be subsequent to, and unrelated to, the cause of NTDs” [64]. Furthermore, the exact positions of open defects may not be interpretable with respect to inferences about locations of closure initiation sites. In mouse models, embryos sharing the same genetic etiology often differ in the exact location of the open neural tube. For example, in the SELH/Bc model, the rostral edge of exencephaly may be located anywhere between the caudal limit of the forebrain and the middle of the midbrain and, independently, the caudal edge of the exencephaly may be located anywhere between the caudal limit of the midbrain and the caudal limit of the hindbrain (Figure 4 in [65]). In the SELH model, we have shown that, in the absence of a midbrain initiation site, the normal closure initiated in the forebrain can compensate by advancing caudally to close the entire midbrain, and that closure of the hindbrain can also compensate by continuing rostrally to close part of the caudal midbrain. The variation in location of exencephaly seems to reflect stochastic variation in the efficiencies of these compensatory processes. We suggest that this type of compensatory mechanism might explain part of the documented variation in locations of human anencephaly [66].

### 3.3. Cell Type of Initial Contact

The tips of the neural folds expose the border between the neuroepithelium and surface ectoderm. The cell type involved in initial contact between apposed folds varies regionally. In the mouse forebrain region, first contact across the gap is between the apposed neuroepithelia, whereas in the midbrain and hindbrain, the initial contact and apparent adhesion is between cells of the surface ectoderm, which in the midbrain wraps around the neuroectoderm as the folds meet so that surface ectoderm faces across the closing gap [37,67]. In the posterior neuropore, the surface ectoderm may make first contact, based on locations of cellular projections [5].

The fusion of the neuroepithelium and the surface ectoderm may be independent; a closed cerebellar defect arising in an unfused neuroepithelium beneath a fused surface ectoderm has been described in mice [65].

### 3.4. Cell Projection Types

Fusions between epithelia in various tissues and species are typically preceded by various cellular projections such as ruffles, filopodia and lamellipodia [38]). Dynamic cell behaviors and interactions, such as the interdigitation of ruffles across the gap between the closely apposed neural folds of the closing neural tube, are seen and the projections are required for neural tube closure [38,55,68]. The density and type of cell projections vary along the anterior–posterior axis of the neural folds [38,55]. The forebrain, midbrain and hindbrain differ in the presence of filopodia and ruffles. In the forebrain region, live imaging of surface ectoderm has shown that there are a few small narrow projections close to the area of contact, but the majority of the surface ectoderm does not have projections [39]. In the midbrain region as it approaches closure, there are many surface ectoderm projections that appear to be filopodia [39]. In the closing hindbrain region there are many long round projections from the surface ectoderm, some of which may be filopodia, and others a relatively narrow form of ruffles [39]. The upper spine Closure 1 initiation region has wide membranous ruffles or lamellipodia and narrow bulbous projections, both extending from the surface ectoderm towards the gap at the midline and increasing as the folds move closer together; these projections are the first points of contact across the gap [39]. The caudally advancing closure region of the upper spine region has filopodia [37]. The mid-spinal region has both filopodia and ruffles in the closing area. The most caudal region of primary neurulation has only ruffles [38,55].

The regional differences in the abundance of filopodia and ruffles reflect, at least in part, differences in regulators of the actin cytoskeleton, *Rac* and *Cdc42* [38]. These GTPases are molecular switches that link extracellular signals to specific downstream effectors. In general, *Rac1* induces the formation of branched actin networks needed for lamellipodia and ruffles, whereas *Cdc42* leads to assembly of unbranched actin bundles that form filopodia [38].

### 3.5. Regional Differences in Ephrins and Ephrin Receptors

The gene families of membrane-bound Ephrin receptor tyrosine kinases (*Ephs*) and their membrane-bound ligands, the *Ephrins*, are involved in cell–cell interactions throughout development. Interaction between *Ephrins* and their receptors may cause cell adhesion or repulsion. They are involved in the adhesion of apposed neural folds for fusion and may have a role in the etiology of NTD. This area has not been deeply studied and roles of more members of these gene families are likely to be identified in future studies. Expression of some combinations of *Ephrins* and *Ephs* may differ between cranial regions and also differ from the caudal region. *EphA4* is expressed in the midbrain region in early neural fold stages [69]; *EphA2* and *EphA4* are expressed in the hindbrain neural folds before closure [70]. *EphrinA5* and its receptor *EphA7* splice forms are expressed in the dorsal cranial neural folds (midbrain and hindbrain) before and during closure, but not in the spinal neural folds [40]. In the posterior neuropore, *EphA1* is expressed in the neural plate and other tissues, *EphA2* in the neural plate and tips of the apposed neural folds, *EphA4* in the mesoderm and tips of the apposed neural folds, and *EphA5* in the neural plate [43]. Further study of *EphA2* in the tips of the neural folds located its expression to surface ectoderm cells and their lamellipodia-like protrusions that extend towards the apposed neural fold [43].

### 3.6. Requirement for Grhl Gene Family Expression

The regionality of the requirement for expression of different members of the *Grhl* gene family along the anterior–posterior axis of the neural folds in mice is an important demonstration that the locations of NTD reflect the regional requirement for expression of specific genes. Loss of function of *Grhl3* causes thoraco-lumbo-sacral spina bifida and curled tail (a microform of spina bifida) in all homozygotes and concomitant exencephaly in a few [71]. Loss of function of *Grhl2* causes split face and open cranial neural tube extending through the hindbrain to the cervical region and, depending on the mutant allele and genetic background, spina bifida [37,41]. Heterozygotes at either locus are normal, but some digenic heterozygotes (*Grhl2*^+/−^;*Grhl3*^+/−^) have lumbo-sacral spina bifida with curled tail or exencephaly and/or curled tail. *Grhl2* null and *Grhl3* null embryos fail to form DLHP in the regions that will have failed neural tube closure [41]. 

Further study [41] indicates that *Grhl2* is essential to closure of the forebrain and cooperates with *Grhl3*, whereas *Grhl3* is essential to closure posterior to the mid-thoracic region, and cooperates with *Grlh2* for closure of the posterior neuropore (PNP). Neither *Grhl* gene is required for closure of the cervical region to the thoracic region, the rostral part of the Closure 1 zone; this is the only region of the neural tube that does not form a concave shape of neural folds before closure, and may therefore be the only region that does not have DLHPs. DLHPs are lacking in the PNP of both *Grhl2* and *Grhl3* mutants; other regions were not studied. In summary, the *Grhl2* and *Grhl3* genes may be respectively essential for DLHP formation in separate zones of the developing neural tube, and may also function cooperatively for DLHP formation in other zones. The *Grhl* mutants did not alter the expression of some genes known to be important in DLHP formation, such as *Bmp2*, *Noggin*, and *Zic2* [41]. The failure to form DLHP may be explained by the observation that *Grhl2*, which is specifically expressed in the surface ectoderm of the cranial neural folds, is required to suppress transformation of ectoderm to mesenchyme [72]. The acquisition of mesenchymal characteristics and loss of integrity of the surface ectoderm likely causes the inability to bend the dorsal neural fold. A similar disruptive effect on the development of DLHPs or the bending to concave shape is suggested for the homolog, *Grhl3*. In the caudal region *Grhl3*, is expressed in surface ectoderm during neural tube closure, and lack of *Grhl3* seems to cause delay of specification of surface ectoderm [73], a situation somewhat like the findings for *Grhl2* [72]. Later, at the most caudal end of primary neurulation, a second mechanism is observed. *Grhl3* is expressed in the gut endoderm that underlies the neural tube, and lack of *Grhl3* expression there is associated with defective hindgut cell proliferation that causes excessive axial bending that mechanically interferes with closure of the posterior neuropore [73].

### 3.7. Dynamics of the Actomyosin Cytoskeleton of the Neuroepithelium

Actomyosin “machinery” (actin filaments, phosphorylated myosin light chain (pMLC) and RhoGTPases) is located circumferentially in the apices and apical junctions of all neuroepithelial cells. Its contraction is thought to be responsible for apical constriction, contributing to neural fold bending, and is required for cranial neural tube closure but not spinal neural tube closure. Mice with mutations affecting actin-associated proteins develop cranial, but not spinal, NTDs. Conversely, the caudal spinal region as well as the cranial region requires actin turnover for neural tube closure and abnormal accumulation of apical actomyosin does cause failure of neural fold elevation in both regions [19]. RhoA and its effector ROCK are required in the neuroepithelium, to maintain balanced apical actomyosin accumulation, to regulate actin turnover, as well as for convergent extension [19,20]. A related caudal difference is the contribution of bipotential neuromesodermal progenitors to the caudal neuroectoderm and somatic mesoderm, and the influence of PCP (*Vangl2*) on their differentiation as well as on neuroepithelial actomyosin contractility [44].

### 3.8. Other Examples: Hox, Neural Crest and Apoptosis

Other regional differences in the neural tube also have an unknown or uncertain role in closure mechanisms. For example, numerous *Hox* genes, which convey anterior–posterior positional information, are expressed in many different combinations along the midline axis before neural tube formation, with the earliest and fewest expressed in the hindbrain region and the latest and most numerous expressed in the caudal spinal region; in contrast, none are expressed in the cranial region rostral to the hindbrain [74,75]. Another example is the timing of emigration from the tips of the neural folds by the neural crest, which in mouse emigrates after closure in the spinal region and before closure in the head, and whose role in neural tube closure is uncertain [42,76]. There also may be a requirement for normal apoptosis in the cranial neural folds to enable closure, but having less effect in the spinal region [42,77]. 

## 4. Evolutionary Aspects

### 4.1. Modern Vertebrates

Most of our understanding of neural tube development is based on a small number of species: mouse, chick, frog (Xenopus), rat, and zebrafish. Among these, there are very important differences in the basic mechanisms by which the neural tube is formed. All likely share the basic planar cell polarity/convergent extension mechanism [5]. Details of convergent extension in development of the neural tube have been studied in frog (Xenopus) and zebrafish, but their cellular process is expected to differ from mammals and chick because their organization of neuroepithelial cells is known to differ [16]. For the hinge point mechanism, Xenopus lacks the MHP and DLHP [20]. Overall, the studied species differ in various mechanisms of closure, such as the relationship of the neuroepithelium to the surface ectoderm, the presence of neural folds, the presence of hinge points and the degree to which closure is intermittent or simultaneous along the whole neural tube [5,78,79,80]. Of course, a species that does not normally undergo the cellular/tissue process whose failure causes human NTD cannot model the mechanism of human NTD. Among the well-studied models for neural tube development, mouse, chick and rat appear to be most similar to human, but little is known about human embryos except histological and morphological studies. Based on morphological studies, the little-studied species, rabbit and pig, may be most similar to human (Figure 3). 

### 4.2. Proxies for the Ancestral Pre-Vertebrate

The Cephalochordate, Amphioxus (lancelet), whose lineage diverged from vertebrates 550–600 million years ago, is used as a proxy for the ancestral chordate [81,82]. Like vertebrates, Amphioxus forms a neural plate, with a neural plate border region. Whereas in the representative mammals studied for neural tube development, the border region remains intact and is carried to the midline with the medial folding of the neural folds, in Amphioxus, the neural plate border cells, using lamellipodia, migrate medially to cover the neural plate as continuous surface ectoderm and beneath it, the neuroectoderm bends to form a tube [81,83]. The Amphioxus genome has been sequenced [83]. *Noggin*/*BMP* signaling and parts of the *Wnt*/PCP signaling pathway are present [81] but a detailed analysis of the pathways known to be important in neural tube formation does not seem to have been done.

The lineage of Tunicate chordates, the Ascidians (sea squirts), diverged from the vertebrate lineage around the same time as Cephalochordates [84]. The larval sea squirt forms a neural plate and neural folds in a manner similar to the representative mammals. The neural plate curls towards the midline to form a tube, pulling with it the adjacent surface ectoderm to the midline, which meets and fuses to form the epidermis that covers the neural tube. The neural plate cells elongate and change from columnar to wedge-shaped during this process, with narrow apices. Closure progresses from posterior to anterior [85]. Nodal signaling may be required for neurulation. Convergent extension is present [86]. It is not clear that the notochord has a role in patterning of the neural tube, unlike in vertebrates [87]. As in vertebrate neuroepithelium, cell cycle alterations in both the surface ectoderm and the neural plate facilitate the bending required for closure. Movement of the midline epidermal cells requires Rho/ROCK signaling and medial actin filament accumulation [87].

The similarities of neural tube development in comparisons of mammals and Amphioxus and Ascidian embryos suggests that the rolling up of the neural plate into a tube is the ancient mechanism, and from Ascidian embryos that the formation of neural folds is also the ancient mechanism. The divergences from this pattern in species such as zebrafish are therefore new evolutionary products. Some of the ancient processes, such as migration of the neural plate border cells to form the surface ectoderm over the tube in Amphioxus suggest the possibility of atavistic capabilities lurking in the mammalian genome, potentially available in some circumstances.

## 5. The Cranial Neural Tube

### 5.1. The New Head and the Cranial Neural Crest

An important context for understanding cranial neural tube closure is that the vertebrate head and its neural tube are considered by the “New Head” hypothesis to be a new structure that was added by evolution, stepwise, onto the basic chordate ancestor which already had the spinal neural tube [88,89]. The genetic basis used to create the New Head includes conservation of early-expressed genes, co-option of other existing genes, and evolution of novel functions for the newly evolved duplicate orthologs of developmental genes [81,82,90]. Consequently, cranial neural tube developmental processes use a blend of old genes and pathways interacting with more recently evolved genes and pathways; processes in the head that may appear to be similar to those in the spinal neural tube may be different at the molecular genetic level.

The ancestral pre-vertebrate chordate representative, Amphioxus, is a filter-feeder with tentacle-like structures around a small mouth, pharyngeal gill slits, a notochord that extends all the way to the front of the head, a neural tube, and a post-anal tail; it does not have jaws, skull, neural crest or ectodermal placodes (optic, otic, olfactory). It has a very simple tiny brain with rudiments of hindbrain, diencephalon, and perhaps some midbrain, but no telencephalon [81]. Amphioxus has some basic ancient conserved gene networks used in development of the neural plate, neural crest and placodes, but not the more downstream components. It does not have a neural crest per se, because genes required for the early development of neural crest cells, although present, are not expressed at the edges of the neural plate [90].

Evolution of a neural crest set the stage for the development of the New Head [91]. Most of the vertebrate face and jaw complex is derived from cranial neural crest cells [92], which arise from the neuroectoderm at the edge of the neural plate and migrate subectodermally ventrally and rostrally. The sensory placodes, another aspect of the New Head, also arise at the edge of the neural plate, induced in the adjacent non-neural ectoderm [89]. In the mammalian head, the neural crest cells emigrate from the neural folds before the folds have elevated; in the spine, they emigrate after closure [60,76]. The neural crest functions differently in the New Head than in the spinal region. For example, in the New Head, cartilage is derived from neural crest cells; this seems to have been enabled by the acquisition of a new cis regulator on the *SoxE* gene that causes its expression in cranial neural crest cells [93]. A consideration for cranial neural tube closure is whether there are differences in gene expression in the cranial neural crest versus the spinal neural crest that precede neural tube closure—if so, these differences have potential to affect the process of closure.

Unlike the rest of the vertebrate neural tube, the anterior forebrain neural folds do not generate neural crest cells, and the neural crest cells of the forebrain mesenchyme migrate in from the caudal forebrain and the midbrain [60,94,95,96]. In mouse embryos, the transcription factor *Tcf7l1* expressed in the anterior forebrain neural folds acts to repress the *Wnt/B-catenin* pathway that otherwise would induce neuroectoderm cells to become neural crest cells [97]. Loss of *Tcf7l1* leads to conversion of the neuroectoderm of the most rostral neural folds to neural crest cells and a subsequent reduction of the rostral forebrain, which if severe is accompanied by exencephaly [97]. Interestingly, in the images published, the midbrain also appears to be reduced.

### 5.2. Absence of Hox Gene Expression

*Hox* genes, some of which are present in Amphioxus, are expressed in the neuroectoderm and paraxial mesoderm of vertebrates and impart anterior–posterior positional identity. Under the regulation of *Fgf*, *Wnt* and other genes, various combinations of *Hox* genes are expressed in the newly formed axial tissues from the primitive streak stage onward [75,98]. In vertebrates, *Hox* expression extends from the embryonic hindbrain through the tailbud; there is no cranial *Hox* gene expression rostral of the border between rhombomere 1 and 2 of the hindbrain [98]. An interesting question is whether the absence of *Hox* gene expression affects the subsequent genetic program determining cranial neural tube closure.

### 5.3. The Prechordal Plate and Cranial Flexure

The prechordal plate [34,99] underlies only the rostral forebrain (the telencephalon and the diencephalon region that will become the optic primordium). Histologically, the prechordal and notochordal plates appear continuous, but are distinguishable [99]. Located immediately below the midline of the neural plate, the prechordal plate functions in similar ways to the notochord, which underlies the rest of the neural plate, producing essential regulatory factors such as *Shh*, but it also differs from the notochord, by producing factors such as *Goosecoid* (*Gsc*), an inhibitor of convergent extension [100] and by not producing others such as *cNot1* [34,101,102]. Thus the basic regulatory mechanism underlying the forebrain neural tube differs from the rest of the neural tube.

Although the prechordal plate is initially located in line with the notochord, it is bent to lie at right angles to the notochord just before and during the time the mesencephalic folds are elevating [34,60,99,103]. Concurrently, and centered on the mid-mesencephalon, the angle of the cranial flexure narrows from 150 degrees to 100 degrees in human, and from 100 degrees to 70 degrees in mouse [60,103]. The increased bend of the mesencephalic neural tube at the time of closure can be hypothesized to be a physical force working in opposition to the elevation of the mesencephalic neural folds, and the relative acuteness of the angle in mouse is notable in this respect. The prechordal plate also takes part in the formation of the oropharygeal membrane, which, beneath the neural tube, temporarily separates the foregut from the stomodeum. The oropharyngeal membrane ruptures around the time the midbrain neural tube is closing [60,61]. It would be interesting to discover whether the rupture has any mechanical effect that offsets the narrowing of the angle of the flexure.

### 5.4. Optic Sulci

A further complexity in the mechanical context of closure of the cranial neural tube is the development of the optic sulci in the forebrain. These begin as indentations in the neuroepithelium and balloon outward toward the surface ectoderm as the forebrain neural folds elevate toward each other, so that the apposed forebrain folds resemble a pair of castanets [104] (Figure 5c in [105] and Figures 19 and 20 in [106]). This contrasts with the relative simplicity of the shape of the spinal neural folds during elevation and leads to the question whether the process of shaping the neuroepithelium to form optic sulci affects the process of neural fold elevation that occurs concurrently. 

### 5.5. Sonic Hedgehog and Cranial DLHP

A *Sonic hedgehog* expression gradient in neuroepithelium from upper spine to lower spine accounts for a correlated inverse gradient of dorsal bending of spinal neural folds during closure, as high levels of *Shh* signaling in the upper spine inhibit formation of DLHP, as discussed in Section 2.5. The levels of *Shh* in the ventral neural tube of the head do not seem to have been compared to those of the upper spine; in published whole mount in situ hybridization images, the intensity in the cranial region seems as high as the upper spine (Figure 4C in [32]), but this is not a quantitative method. The *BMP-Noggin*-DLHP relationship and cell behavior at the cranial DLHP does not seem to have been studied, and it is possible that the mechanisms are not entirely the same as in the spine. Conversely, the induction of exencephaly in response to elevated *Shh* signaling suggests that the relationship of cranial DLHP to *Shh* is similar to that in the spinal region and modulated by the large size of the cranial neural folds, as discussed in Section 2.5.

### 5.6. The Actomyosin Cytoskeleton of the Cranial Neuroepithelium

Mammalian cranial neural fold bending for closure requires the actomyosin cytoskeleton, and mutations in its components or chemical disruption of apical actin microfilaments cause exencephaly (summarized in [19]). Thus, mutations in components such as *palladin*, *vinculin*, *cofilin 1* (*Cfl1*) and *Marcks*, or in genes for actin regulatory proteins such as *Mena*, *Vasp* and *Evl*, or in genes for protein kinases with cytoskeletal influence such as *Abl1*, *Abl2*, *Mapk8* and *Mapk9* all cause exencephaly in mice [19]. In contrast, closure of the spinal region does not require these genes. Therefore, these genes important to the actomyosin cytoskeleton are candidate genes specific to human anencephaly etiology.

### 5.7. Apoptosis in Cranial Neural Folds

Programmed cell death, apoptosis, normally occurs in the neuroepithelium during development of the neural tube [42], with a pattern of areas of particular abundance associated with bending and fusion [107]. Various mouse mutants that demonstrate decreased or increased apoptosis in the neural folds develop exencephaly but not spinal NTD [42]. For example, in mouse embryos that constitutively lack apoptosis, the *Casp3* and *Apaf1* null mutants, the midbrain and hindbrain fail to close but the rest of the neural tube closes normally. Live imaging of the midbrain–hindbrain neural folds undergoing bending and closure in normal embryos show conventional and unconventional apoptotic cells [77]. Conventional apoptotic cells (having caspase activation, followed by shrinkage and fragmentation) are in the boundary region and surface ectoderm at the tip of the neural folds and at the midline before and after the completion of closure. The unconventional apoptotic cells (having caspase activation, but not shrinkage and fragmentation) emerge from the neuroepithelium of the boundary domain and dorsal neural fold and accumulate as the fold bends; they are round, protrude from the surface, remain attached for a time, and then tumble along the surface of the neuroepithelium into the lumen of the neural tube. In *Casp3* and *Apaf1* null mutants, neither type of apoptotic cell is found and the apical bending of the folds towards the midline is severely reduced. The mechanism of the effect of apoptosis on bending is not known.

### 5.8. Mutations in Genes Expressed in the Cranial Neural Crest That Are Associated with NTD

Emigration of neural crest cells from the midbrain and hindbrain open neural folds has been thought to be necessary for cranial neural tube closure [42], but the evidence from mouse mutants is limited. Two genes that cause NTD and are expressed in the cranial neural crest are the transcription factor *Tcfap2a* and the cell adhesion factor *Cdh2* (N-cadherin). In mice on E8.5, *Tcfap2a* is expressed in the cranial neuroepithelium and cranial neural crest; *Cdh2* is expressed in the cranial neural plate. Conditional mutants in which either *Tcfap2a* or *Cdh2* is deleted in the neural crest cells in which *Wnt1* is normally expressed, suggest that some aspect of cranial neural crest abnormality causes midbrain exencephaly (15–20% in *Tcfap2a*, [108]; 100% in *Cdh2*; [109]). Although for both mutants, it appears that exencephaly is due to the absence of the targeted gene’s expression in the cranial neural crest cells, for neither mutant is the mechanism of interference with neural tube closure understood. In the *Tcfap2a* mutants, it may involve the positioning of the neural plate border [108]. A complication of these studies is that *Wnt1*-Cre is expressed in both the lateral non-neural ectoderm of E8.5 cranial neural folds and the neural crest [110]. The potential role of the lateral non-neural ectoderm cells in neural tube closure or its failure has yet to be explored.

It is interesting that whereas 100% of *Tcfap2a* null embryos have split face and forebrain/midbrain/hindbrain exencephaly [111,112], the cranial neural crest conditional null mutants have failure of closure of only the midbrain. Further evidence for neural crest involvement emerges from study of *Cdh2* null mutants rescued from early lethality [113,114], which are 100% exencephalic. On E8.5, the rescued embryos have increased apoptosis in the tips of the cranial neural folds, in what appear to be neural crest cells starting to migrate from the caudal midbrain region [114]. In summary, although abnormality in cranial neural crest cells appears to be the cause of exencephaly in the *Wnt1*-Cre conditional null *Tcfap2a* and *Cdh2* mutants, the mechanisms are unclear. We have found no definitive evidence from mouse mutants for delayed or abnormal neural crest cell emigration as a cause for exencephaly.

### 5.9. Role of Cranial Mesoderm in Neural Fold Elevation

The vertebrate cranial mesoderm has an important role in cranial neural fold elevation. The vertebrate cranial mesoderm, created by gastrulation [115], may have accrued differences from spinal mesoderm as part of the evolution of the New Head. Comparisons of mesodermal gene expression between Amphioxus and vertebrates indicate that expression in late gastrula of the mesodermal patterning genes found in both groups is reorganized in vertebrates, limiting expression of some to the head and others to the body, thus creating a novel type of mesoderm in the vertebrate head [116].

**Figure 4:**
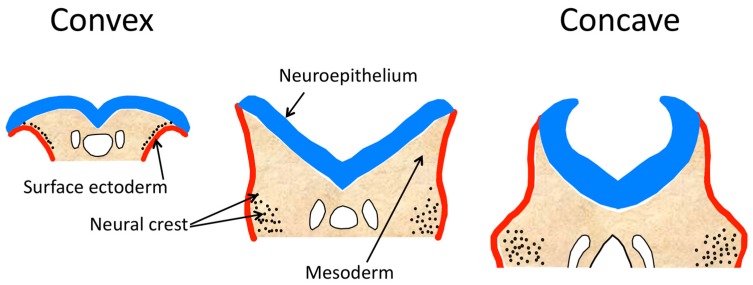
Diagrams of transverse sections of midbrain neural folds during their elevation, showing the morphological change from convex to concave shape. Based on Figure 1 in [117].

The role of cranial mesenchyme (comprised of cranial mesoderm and neural crest cells after their emigration from the neuroectoderm) in the cranial neural tube has been studied in mouse, rat and chick embryos [117]. During cranial neural tube formation, the shape of the folds progresses from biconvex, with the mid-lateral folds cushioned on a large supporting cranial mesenchyme, to biconcave (Figure 4). The neural folds of the spinal region do not have the convex stage. During the process of cranial neural fold elevation, the mesenchyme expands by both increased numbers of cells and increased space between the cells [117,118] and this mesenchymal expansion and reorganization is thought to be important in causing elevation. During the biconvex stage, the neural crest cells leave the neuroectoderm of the lateral tips of the neural folds and begin to migrate subectodermally through the mesenchyme to the first branchial arch and facial prominence areas. As the neural folds elevate, becoming biconcave, and the dorsolateral hinge points form, the mesodermal cells become more widely spaced in the medial region and more closely packed in the lateral region. The extracellular matrix undergoes changes in hyaluronic acid concentration that may provide a mechanism for elevation by mesenchyme expansion, as hydration of hyaluronic acid is known to cause expansion of mesenchyme [117,119].

Several mouse mutants demonstrate the importance of the cranial mesenchyme to cranial neural fold elevation. Loss of function of the transcription factor *twist* causes severe craniofacial defects, including midbrain exencephaly; the neural folds fail to become concave and remain everted [120]. *twist* is highly expressed in the cranial mesenchyme just before and during the time of neural tube closure [121,122]. At the normal time of cranial neural fold elevation, mutant embryos have a morphologically normal neuroepithelium, but have abnormal morphology of mesenchyme cells in the forebrain and midbrain, which are rounded, lack normal stellate shape and have greater intercellular space than normal [120]. The exencephaly is attributable to a defective cranial mesenchyme and not the neuroepithelium, based on chimera studies [120] and on conditional mutants. Ablation of *twist* in the precursors of the cranial mesoderm causes reduced proliferation and abnormal clustering of mesoderm cells in the lateral regions of the head folds and subsequent exencephaly [121]. No evidence of increased cell death has been found [121]. The neural crest seems to form normally [123] and the neural crest cell-based facial abnormalities are considered to be a secondary effect of the abnormal mesenchyme failing to provide essential signals to the neural crest cells as they migrate to the face [121]. Based on studies later in development, the loss of expression of *Cart1* (*Alx1*) and *Alx3* in *twist* null mutant embryos suggests that they are in the same regulatory framework [123], and it is interesting that they also express exencephaly in mutants.

The *Cart1* (*Alx1*) transcription factor is expressed in mesenchyme cells of the forebrain but not the midbrain, and not in neuroepithelium, during neural tube development [124]. Null mutant embryos for *Cart1* (*Alx1*) have increased apoptosis and transient deficiency of mesenchyme in the forebrain, with delayed closure of the forebrain folds; subsequently, the midbrain neural folds fail to elevate, leading to exencephaly [124]. Whereas *Cart1* (*Alx1*) early expression may be limited to the forebrain mesenchyme, a homolog, *Alx3*, appears to be expressed in paraxial mesoderm of the body and the mesenchyme of the head, but also not in the neuroectoderm, at the time the cranial folds are elevating [125]. Although some authors assume *Cart1 (Alx1)* and *Alx3* expression in the neural fold mesenchyme is in the neural crest cell component, this seems unproven. About 10% of mutants lacking *Alx3* develop midbrain exencephaly and/or split face (failure of closure of the forebrain neural folds); the neural folds of these embryos fail to undergo the shift from convex to concave or development of a dorsolateral hinge point and therefore fail to meet in the midline [126]. Increased apoptosis and decreased mesenchymal cell density in mutants are present in later developmental stages [126], but is not known if any abnormalities in apoptosis and mesenchymal cell density are present earlier when they could contribute to the failure of neural tube closure. Interestingly, *Alx3* expression is lost during folic acid deficiency at the time of cranial neural tube closure, whereas expression of other neural tube genes such as *Cart1 (Alx1)*, *twist*, and *Cited2* is not affected [126].

*Inka1* is first expressed in the cranial mesenchyme prior to and during neural tube formation and then in the cranial neural crest during its migration in the mesenchyme [127]. It is not expressed in the neuroepithelium. The *Inka1* protein inhibits the *Pak4* kinase that may function in cell–cell contacts and adherent junction formation [128]. *Inka1* is regulated by *Tcfap2a* in non-mammalian vertebrates, but not in mice [127]. Loss of function of *Inka1* causes a predisposition to midbrain exencephaly (5–7%); otherwise, individuals are normal and fertile. The mechanism by which the neural tube fails to close has not been demonstrated, and the mechanistic role of the mesoderm or neural crest or both is unknown.

Loss of function of the transcriptional regulator, *Ski*, [129] which causes exencephaly or split face [130], is sometimes listed as having abnormal cranial mesenchyme [117] but cranial tissues do not seem to have been studied during the period of cranial fold elevation [130,131], and an etiology of exencephaly based on effects on cranial mesenchyme seems unproven.

Null mutant embryos for *Smarca4* (*Brg1* or *SW1/SNF*), involved in chromatin remodeling, die before implantation. Heterozygotes often have midbrain exencephaly, and no other defects [132]. *Smarca4* is expressed strongly in the cranial mesenchyme before elevation [133]. The mechanism causing the failure of neural tube closure has not been studied.

Mutations causing loss of function of the E3 ubiquitin ligase *Hectd1* (*opm*) lead to midbrain exencephaly in all homozygotes and some heterozygotes [134]. *Hectd1* is ubiquitously expressed in embryos, including the head mesenchyme, and the cause of exencephaly is attributable to the effects of *Hectd1* deficiency on the cranial mesoderm cells, which are abnormally shaped and packed more densely than normal near the neural tube at the normal time of elevation, and which fail to orient parallel to the neuroepithelium [117,134]. The neural folds lack mesenchyme expansion and remain flat or convex and fail to form the dorsolateral hinge points. The neural crest cells’ migration pattern and differentiation are normal; mesenchymal cell proliferation and apoptosis are normal [134]. Before the normal time of elevation and closure, *twist* has an expanded area of expression in the midbrain folds in the *Hectd1* mutants, and after failure of full elevation, *Snail* and *Pdgfr* are abnormally expressed in the dorsal area of the midbrain folds [134]. One of the *Hectd1* substrates, heat shock protein 90 (*Hsp90*), is known to enhance migration in various cell type, is secreted at elevated rates from *Hectd1* mutant cranial mesenchyme cells which are abnormally migratory in vitro [135]. It seems that the effects of the lack of *Hectd1* in the midbrain neural folds lead to excess *Hsp90* secretion, which in turn alters the behavior of the mesoderm cells, leading to their failure to contribute to elevation of the folds.

Loss-of-function mutants for the *Cecr2* chromatin-remodeling factor develop exencephaly [136]. *Cecr2* is expressed in neuroepithelium and mesenchyme during cranial neural tube development [137]. Studies of gene expression arrays from mutant embryos show reduced expression of *Cart1*/*Alx1* transcription factor [138]. As this mesenchymally-expressed gene is required for cranial neural tube closure [124], it appears that the defect in *Cecr2* mutants may be attributable to defective cranial mesenchyme. The observation that in *Cecr2* mutants, the cranial neural folds do not elevate sufficiently for contact between the apposed folds, despite forming DLHP [137], is consistent with the hypothesis that the NTD is caused by an insufficient expansion of the cranial mesenchyme to assist elevation. 

Exencephaly in mouse mutants is usually caused by a failure of midbrain neural fold elevation and it appears that defects in cranial mesoderm are a common etiology for this defect. Exencephaly becomes anencephaly, owing to degradation of exposed neural tissue during later gestation, and therefore is seen as anencephaly at birth in humans [6]. Interestingly, as in the mesodermal mutants in mice, unelevated everted midbrain neural folds have been observed in some early NTD human embryos [139,140], suggesting that lack of elevation owing to mesodermal defects also could be a common mechanism in human anencephaly.

### 5.10. Neuroepithelium-Expressed Genes and Mechanisms That Cause Exencephaly

The actomyosin-dependent shaping and bending of the cranial neural folds is based in the neuroepithelium, and it seems predictable that the many actomyosin-related genes that cause NTD would show neuroepithelium-specific expression, but data to demonstrate this is lacking. Many studies report whole mount in situ hybridization in the cranial neural folds without demonstration of the cell type involved (neuroectoderm, mesenchyme, surface ectoderm). A few genes that cause cranial neural tube defects when dysfunctional have been reported to be expressed in the neuroepithelium.

*Cfl1* (*Cofilin 1*, *Cofilin-n*) is expressed in the pre-closure cranial and caudal neuroepithelium and neural crest [141,142]. *Cfl1* enables the dynamic reorganization of the actin cytoskeleton by its function in F-actin, severing and depolymerization and by promoting the recycling of monomeric actin [141]. Mutants have exencephaly and delayed closure of the posterior neuropore (PNP) [19,141]. Originally, the exencephaly was ascribed to a demonstrated lack of neural crest cell migration, but no mechanism for the lack of neural fold elevation was demonstrated [142]. More recent work has shown that the mechanism causing the exencephaly is a lack of activation of apical actomyosin in neuroepithelial cells and abnormal basal accumulation of F-actin [141]. Consistent with the different roles of actomyosin in cranial versus caudal neural folds, the delayed PNP closure of *Cfl1* mutants is owing to actomyosin accumulation, lack of F-actin turnover and actomyosin disassembly [19].

*Fat1* cadherin, a large cell adhesion molecule, is expressed in the early anterior neural plate in the forebrain and midbrain neural folds during neural fold elevation and in the caudal neural folds [143]. It seems to be located in the neuroepithelium and neural crest. *Fat1* is thought to regulate actin cytoskeletal organization at cell peripheries. In cell cultures, it is found in filopodia and cell–cell boundaries, overlapping with dynamic actin structures; it regulates actin polymerization [144]. Therefore, the cause of the NTD is likely a deficiency of neural fold bending. On some strain backgrounds, *Fat1* null mutants have exencephaly, but not spina bifida. Although mutations of the family member *Fat4* do not cause NTD, and *Fat4* binds different actin-regulating and junctional proteins than *Fat1*, they form heterodimers, and on some strain backgrounds, the combination of homozygous null mutations at both *Fat1* and *Fat4* cause increased frequencies of exencephaly compared with *Fat1* alone [145]; this is an example of the genetic complexity of NTD etiology. 

*Cited2* (*Mrg1*) is a transcription regulator that serves as a co-activator for several transcription factors such as *Tfcap2*, *Smad4*, *Lhx2*, *Nanog* and *Tbx3* [146,147,148]. It is expressed during neural fold elevation in the midbrain neuroepithelium and in migrating cranial neural crest [146,149]. Null mutants have exencephaly. The mechanism leading to exencephaly is likely the disrupted integrity of the neuroepithelium, owing to the observed intense localized apoptosis in the midbrain neuroepithelium, particularly at the midbrain–forebrain boundary, at the time of normal midbrain-fold bending [146].

Members of the *Sall* gene family *Sall1*, *Sall2*, and *Sall4* are transcription repressors that interact with chromatin remodeling complexes, can form heterodimers [150] and are all expressed in the cranial neuroepithelium during neural fold elevation [151,152]. Loss-of-function mutants for each of these *Sall* genes cause exencephaly, depending on strain background [150,152,153], and digenic heterozygous mutant combinations of *Sall1* with *Sall4* or *Sall2* with *Sall4* also cause exencephaly. Based on embryos with a combination of null *Sall1* and hypomorphic *Sall4*, it appears that the cause of exencephaly is a deficiency of elevation of the cranial neural folds, which do elevate part way but do not reach approximation in order to fuse [152].

Members of the *Nuak* gene family, *Nuak1* and *Nuak2*, are serine-threonine kinases that have many functions [154]. *Nuak1* is expressed in many embryonic tissues including the neuroepithelium, but null mutants do not have NTD; *Nuak2* is specifically expressed in the entire neuroepithelium during neural tube closure, and some of the null mutants have midbrain and hindbrain exencephaly; digenic null mutants for both genes all have exencephaly of the forebrain, midbrain and hindbrain [154]. The cranial neural folds in the digenic mutants seem to lack DLHP, and the cause seems to be a significant lack of apical constriction of the cranial neuroepithelial cells during the normal elevation stage [154], which would be expected to cause a lack of bending.

*Shroom3* (*Shrm*), encodes a cytoskeletal protein that binds F-actin and regulates its distribution within the cell. During neural fold elevation, *Shroom3* is strongly expressed in the cranial neuroepithelium, rostral to the otic vesicle, but not in the adjacent cranial mesenchyme [155]. Later, it is expressed in the caudal neural folds at the posterior neuropore during elevation (gene k8220b03, Appendix, and Figure 1 in [156]). All *Shroom3* mutant homozygotes have midbrain and hindbrain exencephaly, most also have split face (failure of forebrain neural tube closure), and some also have spina bifida aperta. The morphology of the neuroepithelium of the closed spinal neural tube is also abnormal [155]. In *Shroom3*-mutant embryonic neuroepithelial cells, F-actin is shifted away from the apical surface, whereas in wild-type cells, it is predominantly localized at the apical surface of the cells. This pattern suggests that the mechanism of the neural tube closure defects in *Shroom3* mutants is the lack of actomyosin-dependent apical constriction in the neuroepithelium. There is evidence, based on various digenic heterozygous mutants involving *Vangl2* (*Lp*) and *Shroom3*, that the actomyosin system is linked to the PCP-convergent extension system by *Shroom3* [157]. For example, whereas neither *Shroom3/+* nor *Lp/+* mutants have spina bifida aperta, the digenic mutants (*Shroom3/+*, *Lp/+*) have almost 40% spina bifida aperta. At the molecular level, there is evidence that that a PCP factor (*Dvl2*) determines the location of *Shroom3* at mediolateral cell junctions along with F-actin, *Rock1* and *Myosin IIb*, components of the actinomyosin pathway [157], suggesting that *Shroom3* and *Dvl2* may interact to link PCP signaling to actomyosin contractility. This is an example of how simultaneous reduction in a genetic component of each of two pathways can cause NTD.

### 5.11. Cranial Ephrins and Ephrin Receptors

An example of the potential molecular complexity underlying neural tube fusion and leading to a cranial-specific etiology of an NTD is an apparent regulatory relationship between the transcription factors *Dlx5* and *Msx2* with each other and with the downstream membrane-bound cellular adhesion/repulsion factors *EphrinA5* and *EphA7. Dlx5* is expressed in the dorsal tips of the neural fold before, during, and after neural tube closure, extending from the forebrain to the posterior neuropore [158,159], and null mutants have some (19–28%) exencephaly [159,160]. Similarly, *Msx2* is expressed before closure in the dorsal edges of the neural tube, except in the forebrain region [161,162], but null mutants do not have NTD [160,163]. In contrast, double mutant embryos null for *Msx2* and *Dlx5* have a high frequency (73%) of exencephaly that seems to be caused by their joint regulatory effect on the splicing of *EphA7* transcripts [160]. The ligand *EphrinA5* and its receptor *EphA7* splice forms are expressed in the dorsal cranial neural folds (midbrain and hindbrain) before and during closure [40]. The interaction of *EphrinA5* with full length *EphA7* causes cellular repulsion, but a co-expressed alternative splice form of truncated *EphA7* converts the effect of the interaction to adhesion [40]. *EphrinA5* null mutants sometimes develop exencephaly (17%), a few of which have failed forebrain closure as well as midbrain and hindbrain, having failed to fuse despite elevating to become juxtaposed at the midline; some (25%) *EphA7* null mutants also have exencephaly [40]. Null mutants for either *Msx2* or *Dlx5* have fairly normal expression of *EphrinA5*, full-length *EphA7* and truncated *EphA7* in the cranial neural tube. In contrast, in *Dlx5/Msx2* digenic null mutants during the stage of neural tube closure, the expression of *EphrinA5* at the apex of the neural folds is decreased, full length *EphA7* expression is unchanged but the truncated form of *EphA7* is greatly reduced, a situation that is expected to cause repulsion, rather than adhesion of the apposed midbrain and hindbrain neural folds. It appears, therefore, that the mechanism leading to exencephaly is likely lack of adhesion of neural folds in each of the *EphrinA5*, *EphA7* and digenic null *Msx/Dlx5* mutants, and the limitation of the NTD to the midbrain and hindbrain reflects the limited expression domain of *EphrinA5* and *EphA7*.

Demonstrating another kind of complexity, null mutation carriers for the X-linked gene, *EphrinB1*, express exencephaly at a higher rate in heterozygous females than in homozygotes and null males [164]. Embryos have not been studied during the period of cranial neural tube closure, but the mechanism may be segregation of *EphrinB1*-positive and -negative cells in the neuroepithelium, affecting its structural integrity [164].

### 5.12. Cranial Neural Fold Projections

In general, it seems likely that genetic defects whose mechanisms cause cranial neural tube defects through failure of fusion of apposed neural folds, would demonstrate defects in the surface ectoderm projections normally present at the fusion sites. However, it appears that data is lacking in this area.

Loss of function of Myosin-X (*Myo10*), an unconventional myosin known for its roles in formation of filopodia and localization to the tips of filopodia, causes exencephaly, along with other non-neural-tube defects [165].

### 5.13. The Phenomenon of Sex Ratio Distortions in Cranial NTD

A striking predominance of females in cranial neural tube closure defects has been recognized for many years; about two-thirds of human anencephalics and mouse exencephalics are female (reviewed in [166]). Human craniorachischisis may also exhibit an excess of females; no sex data are available for mouse mutants with craniorachischisis [166]. Spina bifida as a whole generally does not exhibit female excess in humans or mice [166], although the subtype located in the upper spine may do so [167]. Previously, 12 mouse mutants and strains for which sex had been reported showed an excess of females among exencephalics [166]. There is similar data for two additional mutants: *Cecr2* with 66% females among exencephalics [168], and *Scarb1 (srb1)* with 72% females among exencephalics [169]. The 14 mutants and strains represent a diversity of functions and pathways, suggesting that the increased liability of females to cranial NTD extends across a wide range of causal molecular mechanisms. There may be exceptions. For example, the lack of female predominance in NTD caused by mutations at *Gldc* [170] and *Cited2* [146] requires further confirmation of sex ratios with larger samples. If other exceptional mutants that lack female excess in exencephalics can be found, a comparison of the function of genes with female excess with those without might point to the mechanism responsible for elevated female susceptibility to cranial NTD.

The causes of the female excess among cranial neural tube closure defects in humans are difficult to identify, but in mice, several of the possible explanations have been ruled out by the data. For example, the neural tube develops long before gonadal hormones can be present, the developmental rate of the cranial neural tube during closure does not differ between males and females, and there is no evidence of preferential death of affected males during gestation [166]. In mice, genetic experiments have demonstrated that the higher susceptibility to cranial NTD is caused by the presence of two X chromosomes in females, not the absence of the Y (reviewed in [166]).

There is an intriguing phenomenon of methylation differences correlated to number of X chromosomes in mouse and bovine embryos, during the early developmental period when both X’s are normally active, such as in blastocysts or embryonic stem cells. The global DNA is hypomethylated and the *Dnmt3b* (*DNA methyltransferase 3 beta*) transcript and protein levels are reduced in XX embryos relative to embryos with one X [171,172]. Whether this phenomenon has later consequences affecting gene expression in neurulation stage embryos is unknown.

In mammals such as humans and mice, males have one large X chromosome with over a thousand genes and one small Y chromosome, which contains, amongst a few genes, the *Sry* testis-determining gene; females have two copies of the large X, of which one copy has been mostly silenced beginning from about the time of implantation [173] by a combination of epigenetic changes including DNA methylation, histone modifications and chromatin remodeling [174]. Chromatin (DNA and its attached histones) that has undergone transcriptional silencing and remodeling to become densely packed is termed “heterochromatin”.

A mechanism that has been suggested to explain a variety of disorders that occur at higher frequencies in females than in males is that some genes on the inactive X variably escape from silencing and cause a gene expression imbalance; there is evidence in support of this mechanism for the autoimmune disease lupus erythematosus [174].

Another hypothesis to explain the female excess is that the use of cellular resources to inactivate a large X chromosome in females after every mitosis would competitively reduce the availability of those resources for gene regulation on the autosomes, weakening the process of neural tube closure [64]; updated in [166]. A similar hypothesis has emerged from genetic studies in Drosophila, the “heterochromatin sink” [175]. In Drosophila, a chromosome with a large heterochromatin component can sequester various limited factors required to regulate genes, and this can alter the expression of genes in the rest of the genome [175]. Some observations suggest that the large inactive heterochromatic X chromosome in female mammals can act as a sink that sequesters epigenetic factors that are present in limited amounts (e.g., DNA methyl transferases and histone modifying enzymes), thus altering their availability in the rest of the genome. A mutation at *Smchd1*, a gene involved in the methylation of DNA (formerly *Momme D1*) causes reduced methylation and silencing of a reporter transgene and autosomal retrotransposons, but only in females. The female-specific effects led Blewitt et al. [176] to suggest that the inactive X chromosome could be sequestering proteins that are involved in controlling gene expression at autosomal loci. An example of this phenomenon has been demonstrated by the “four core genotypes” system, where the male sex determining factor *Sry* has been moved to an autosome, comparing XX females, XX males, XY males, and XY females. It shows that the expression level of hundreds of genes in mouse thymus cells is determined by the number of X chromosomes present and not by gender [177]. Genes differ by whether they are sensitive to the number of X chromosome and in degree of sensitivity [177]. The sensitive genes have lower expression in the presence of two X chromosomes [177]. If some of the important genes that have cranial-specific neural tube expression (Section 5) are relatively sensitive, and if there are not sensitive genes among the important spinal-specific neural tube genes, this would explain the female excess in cranial, but not spinal, NTD. Perhaps examination of expression levels of genes in the neural folds of embryos from this “four core genotypes” system would yield insight into the causes of female excess in cranial NTD.

There is an interesting interaction between embryo gender and the preventative effects of folate on human NTD. In at least three populations from Chile, Argentina and Canada, the female excess among anencephalic births disappeared soon after the start of folic acid fortification of wheat flour [167,178]. Prior to folate supplementation, anencephalic births in each country exhibited the typical excess of females, where almost two-thirds of anencephalic births were female. Fortification with folic acid reduced the occurrence of anencephaly in both males and females, but had a greater effect in females; for example, 50% prevention in males versus 70% in females [167]. One way to interpret the pattern would be that the additional folic acid compensates for the “heterochromatin sink” in females and restores availability of essential chromatin regulatory factors, reducing female risk of NTD to the same level as males. Then, through perhaps a different mechanism, the abundant folate interacts with a particular type of genetic weakness present in a subset of cases to reduce NTD risk in that subset equally in both sexes.

## 6. The Spinal Region, PCP and Craniorachischisis

Much of the understanding of basic mechanisms of neural tube development is based on studies of the spinal region, and spinal neural tube closure has been well reviewed by others [5,6]. We have considered the basic mechanisms (Section 2) and differences between cranial and spinal regions (Section 3). In this section we consider the NTD that is caused by failure of closure of the entire spinal region. The first initiation site for neural tube closure is in the upper spine. There the folds are brought together by, in part, the combined effect of convergent extension and the medial hinge point (MHP, Section 2.3). 

The failure of convergent extension (Section 2.2) results in a short body axis and broad neural plate, with no medial hinge point (MHP), placing the lateral neural folds too far apart to meet in the midline and fuse [179,180]. This results in the NTD, craniorachischisis, in which all but the most rostral zone of primary neurulation has failed and the neural tube remains open from the midbrain or hindbrain, along the length of the spine to the lumbar region in humans and to the sacral region in mice. It is interesting to note that the caudal limit of primary neurulation is more rostral in humans than in mice [46]. The constant feature of craniorachischisis, a closed neural tube in the most rostral, forebrain region, is likely explained by Ulmer et al. [100], who show that *Goosecoid* (*Gsc*), present in the prechordal plate and not in the notochord, appears to repress convergent extension; i.e., because of *Goosecoid*, normally there is no convergent extension in the forebrain. The role of a disruption in the planar cell polarity (PCP) pathway and the consequent lack of convergent extension in causing craniorachischisis have been well reviewed recently [5]. 

In mice, among the numerous mouse gene mutations that cause NTDs, those that produce craniorachischisis are all components of the planar cell polarity pathway, including the PCP core factors, *Vangl2*, *Celsr1*, *Dvl1/2/3* and *Scrib*; and the less central factors, *Ptk7*, *Cdx1/2*, *Sec24b*, *Sfrp1/2/5* and *Smurf1/2* [10].

In humans, craniorachischisis is a relatively rare NTD for which the only known genetic cause is mutations in PCP genes [181]. The developmental morphology and resultant NTD (wide neural plate and wide open neural tube, except for the closed forebrain) is similar in humans and in mice. Histological study of human embryos with craniorachischisis revealed that they have cervical notochord duplication, side by side, beneath the open neural tube, and a broad *Sonic hedgehog* expression domain; if the neural tube in these embryos appeared closed caudal to the notochord duplication, two parallel neural tubes side by side were found housed in a single dural tube [182]. In the homozygous *Lp* mouse mutant at the *Vangl2* gene (*Vangl2-Lp/Lp*), the notochord at the time of initiation of neural tube closure is loosely organized and comprised of two or three parallel longitudinal structures [179]. The process of convergent extension has been shown to be cell-autonomously defective in both the notochord and neural plate of these homozygous embryos [180].

It is becoming clear that PCP genes can have roles other than in convergent extension that can influence neural tube closure. One possibility (for spina bifida) is that caudal neural tube closure is delayed in some PCP-mutant heterozygotes, as has been observed in the mouse heterozygous *Vangl2-Lp* mutant that has 2% spina bifida [183]. A second possibility, which applies more to the cranial region than the spinal region, is a relationship between PCP and cilia that can cause exencephaly in some PCP mutants. Numerous mouse mutants for genes involved in ciliogenesis have exencephaly [13], including mutants for the PCP factor *Inturned* (*Intu*) with 30% exencephaly [18]. A third possibility arises from observations of interaction between the PCP pathway and the regulation of actomyosin dynamics. In several mutants, the PCP gene involved has been shown to have a role in both convergent extension and actomyosin dynamics [19]. Conversely, the actin-binding protein, *Shroom3*, has been found to interact with PCP pathway proteins during neural tube morphogenesis in mice [157]. PCP gene mutations probably contribute to risk of human anencephaly and spina bifida, albeit in a very small proportion of cases [10]. 

## 7. Studies of Digenic Mechanisms of NTD Involving PCP Gene Mutations

In mice, a large and growing number of digenic mutants, heterozygous for mutations at two gene loci, have been observed to have NTD. The molecular mechanisms by which these digenic or oligogenic combinations lead to NTD are largely unknown, but likely include both cumulative effects along the same pathway or deficiencies in interacting pathways. The known digenics involve at least twenty different kinds of genes [3,10,12], but the extensive study of the planar cell polarity (PCP) system has generated numerous well-studied examples, which provide insight into mechanisms of the genetic complexity of human NTD. Most of the examples at hand involve two genes in the PCP pathway, many involving interactions between heterozygosity for both the *Vangl2* mutant, *Lp*, and one of several other PCP genes. In addition to the PCP–PCP gene interactions, there are digenic mutant examples of PCP genes interacting with genes in actomyosin dynamics, as in *Vangl2*;*Cfl1* [184] and *Vangl2*;*Shroom3* [44,157].

Mouse PCP-mutant studies focused on the NTD craniorachischisis demonstrate the genetic complexity of the role of PCP genes in craniorachischisis [10]. In some examples, mutants are homozygous at only one PCP gene (e.g., *Vangl2*, *Celsr1*, *Scrib* or *Sec24b*) and all embryos have craniorachischisis; heterozygotes do not. Although virtually all mouse mutants with craniorachischisis have mutations in genes of the PCP pathway, often mutations at two or more PCP genes are involved. In one type of digenic example, mutants are homozygous at both of two homologous PCP genes with functional redundancy (e.g., *Dvl1*;*Dvl2* or *Fzd3*;*Fzd6)* and nearly all embryos have craniorachischisis; homozygotes for any one of these genes do not. In yet another type of digenic cause, high frequencies of craniorachischisis are found in mutants that are double heterozygotes for mutations at two PCP genes of different gene families (e.g., *Vangl2*;*Celsr1*, *Vangl2*;*Dvl2*, *Vangl2*;*Scrib*, or *Celsr1*;*Scrib)* [10,185]. It is notable that several of the digenic mutant combinations that cause craniorachischisis also produce some normal, fertile, double heterozygotes. 

Craniorachischisis is not the only NTD caused by PCP mutations in mice. Mutations of various single PCP genes or digenic combinations of PCP genes do not cause craniorachischisis, but do cause the more common types of NTD (exencephaly or spina bifida [10]); several others cause craniorachischisis or exencephaly or spina bifida in different individuals. For example, only exencephaly is found in homozygous single mutants at *Fzd3*, *Intu* or *Fat1*, or digenic mutants at *Vangl2*;*Fzd1* (both heterozygous) or *Vangl2*;*Cthrc1* (heterozygous; homozygous). Only spina bifida is found in digenic heterozygotes at *Vangl2*;*Ptk7*, *Vangl2*;*Grhl3*, *Vangl2*;*Sec24b*, and *Celsr1*;*Ptk7* [10]. Finally, different individuals can have craniorachischisis or exencephaly or spina bifida in digenics heterozygous at *Vangl2*;*Scrib* or *Celsr1*;*Scrib* [185].

The mechanisms by which these non-craniorachischisis defects of neural tube closure are caused by PCP pathway mutations are largely unknown. However, the clear differences known for neural tube formation between anterior–posterior regions (discussed above) raise the possibility that the causal mechanisms involve the ciliogenesis, actomyosin, and microtubule functions of PCP genes, in addition to their convergent extension role (Section 2.2 and Section 6). Effects of the *Wnt*-PCP pathway on the caudal neural tube that cause spina bifida include effects on cell differentiation, cell shape, and actomyosin contractility [44].

Mouse mutants support the digenic (or oligogenic) hypothesis for human craniorachischisis and, further, the hypothesis that digenic heterozygotes for PCP genes can also cause risk for human exencephaly and spina bifida. Human NTD cases of various types have been screened for rare deleterious variants in several PCP genes. Human craniorachischisis is relatively rare and few studies are of craniorachischisis only. Most variant screening studies include exencephaly, spina bifida and other neural tube abnormalities [10]. Among craniorachischisis cases, about 20% have potentially deleterious variants in single PCP genes (*VANGL2*, *CELSR1*, *SCRIB* or *DACT1*) [181,186,187]. The mouse digenic PCP mutants support the suggestion that these cases may be undetected double heterozygotes for PCP mutations. Recently, six human NTD cases digenic for PCP variants were reported as follows: three spina bifida and one anencephaly digenic at *CELSR1*;*SCRIB*; one anencephaly digenic at *CELSR1*;*DVL3* and one spina bifida digenic at *PTK7*;*SCRIB* [188].

Overall, the mouse digenic PCP mutants support the strategy of screening cases with anencephaly, spina bifida or craniorachischisis for PCP gene mutations and, given the usual observation of human heterozygosity for the PCP mutations found, strongly encourage the search for second gene locus mutations in these NTD cases.

## 8. Folate and NTD

### 8.1. Human NTD Prevention by Folate

In addition to genetic susceptibility, environmental factors contribute to risk of developing NTD, including various aspects of diet and maternal health [11,189]. One of the important factors is supplementary maternal peri-conceptional intake of folic acid, which has been demonstrated to reduce the occurrence of human NTD by as much as 70% [190,191] and has led to government policies of fortification of various foods with folic acid in some countries [192]. There is a significant residual, perhaps a third, of NTD cases that appear to be “folate unresponsive” [193]. The mechanism of beneficial effects of supplemental folate on human NTD occurrence is unknown. A paradigm of “folate deficiency” is widely assumed, but the data suggest this concept may be an over-simplification [7]. Although in the absence of folic acid supplementation, risk of NTD appears to increase with lower maternal blood folate levels on average [194], the levels are generally in the normal range, not deficient [195]. In animal models, dietary folate deficiency seldom, if ever, causes NTD [189,196,197]. A hypothesis that supplemental folate compensates for a genetic weakness in the folate metabolic pathway is long standing [194,195]. The folate metabolic pathway is well known and frequently reviewed [7,198]. The response to folic acid has directed the search for genes involved in the etiology of human NTD to focus on the genes that affect folate availability to cells and the genes in the known folate metabolic pathways, such as those involved in DNA and protein methylation and in DNA and RNA synthesis [11,199,200]. Despite extensive studies, in general this approach has not discovered genetic variants with major roles in the etiology of human NTD.

### 8.2. Mouse NTD Prevention by Folate

In parallel to humans, in mice, mutations in most folate metabolic pathway genes that have been tested do not cause NTD [201]. A small proportion of the almost 300 mouse gene mutations that cause NTD have been tested for response to folate supplementation. There are a few mouse mutants whose NTD frequency (usually exencephaly) is reduced by maternal folic acid, and most have no known defect in folate metabolism [201]. Examples are the loss of function mutants for transcription factors *Cart1* and *Cited2*, the histone actetyltransferase *Gcn5*, and the “*Crooked*” gain-of-function mutation of the transmembrane co-receptor for *Wnt*, the *Lrp6* gene [202]. Interestingly, the loss-of-function mutation of *Lrp6* also causes exencephaly and spina bifida but responds to maternal folate supplementation with *increased* frequency of these NTDs [202]. These mutants indicate that the NTD preventative effects of folate are not necessarily through compensation for a deficiency in the folate metabolic pathway [201].

The mutant genes in folate-responsive mouse NTD models may have unexpected connections to folate pathways. An example is the *Pax3* transcription factor mutations [203,204], which have several effects on neural tube cell functions and whose homozygous “*Splotch*” mutants have spina bifida, exencephaly, or both. These mutants have a defect in the folate metabolic pathway that leads to deficiency of de novo pyrimidine biosynthesis; this defect seems to be ameliorated by folate supplementation, which reduces the frequency of NTD by about 40% [203,204]. 

### 8.3. A Possible Effect of Folate on Cilia

A recent study has suggested that folate deficiency or lack of the folate-dependent methylation process may exert adverse effects on neural tube development through an effect on formation of primary cilia [205]. In Xenopus embryos, mouse embryos and various types of cell cultures, folate deficiency, inhibition of methylation, or loss of the reduced folate carrier *Slc19a1* each causes reduced cilia formation, indicating that the folate and methylation pathways are required for cilium formation. The lack of cilia is attributable to the demonstrated deficiency of methylation of *Septin2*, a cytoskeletal protein whose methylation state affects whether it contributes to cilia formation [205]. Cilia are required for normal functioning of the *Hedgehog* signaling pathway [13]. An indicator of the functioning of the *Hedgehog* pathway, the truncated form of Gli3 (Gli3R), is decreased in mouse embryonic fibroblast cells lacking folate or methylation, indicating that the lack of cilia in these cells leads to dysregulation of the *Hedgehog* pathway [205]. Cilial structural defects often cause over-expression of the *Sonic hedgehog* signaling pathway in the developing neural tube, which in turn interferes with dorsal bending of the neural folds and causes exencephaly or spina bifida [13] (Section 2.5). In summary, folate status may affect cytoskeletal regulation, cilia formation and *Hedgehog* signaling. 

*Folr1* (*Fbp1*) is a membrane protein that takes folate into cells. Normally, *Folr1* in mouse embryos is expressed in the neural folds of the upper spine just before and during closure and expression expands bidirectionally along the anterior–posterior axis with advancing closure; it is also expressed in the cranial neural folds just before and during closure, similarly expanding with the advancing closure; it is not detected in the forebrain region and posterior neuropore [206,207]. The *Folr1* null mutant is lethal before neural tube closure, but with maternal folate supplementation, null *Folr1* mutant embryos survive to express NTD, particularly exencephaly [208]. *FOLR1* gene variants do not seem to be important in human anencephaly [209] nor in spina bifida, although *FOLR2* and *FOLR3* may have a small role [210]. A functionally related gene that also causes exencephaly in mice is *Lrp2*, a multi-ligand membrane receptor that seems to mediate uptake of folic acid into cells by *Folr1* at the apical surface of the developing neuroepithelium [211]. Maternal injection with folic acid during early gestation reduces the NTD frequency in *Lrp2* mutants by half; dietary supplementation with folic acid is not effective [212]. 

Cellular folate deficiency may not be the mechanism by which mutants for *Folr1* and *Lrp2* cause exencephaly. There are other mechanisms of uptake of folate into cells that may be more efficient than *Folr1*, such as the reduced folate carriers [200]. In this light, it is notable that there is evidence that *Folr1* can also function as a transcription factor in mammalian cells, with a large number of developmentally important genes as its target [213]. Furthermore, recent observations in *Xenopus* have pointed to a novel mechanism for the role of *Folr1* and folate in neural tube closure [214]. Knockdown of *Folr1* during neurulation in Xenopus causes failure to develop a neural tube. In Xenopus, *Folr1* is expressed at the apical surface of neural plate cells, and interaction between *Folr1* and folate in the medial neural plate is required for these cells’ apical constriction and the bending of the neural plate. *Folr1* co-localizes and interacts with *C-cadherin* and *beta-catenin* in the medial neural plate cells. Apical constriction requires internalization of apically localized *C-cadherin* by endocytosis; in *Folr1* knockdown neural plates, there is a decrease in *C-cadherin*–containing endosomes. Overall, the observations indicate that the primary function of *Folr1* interacting with folate in Xenopus neural tube development may not be related to its functions in metabolism, but rather as a regulator of the cytoskeleton and cell adhesion remodeling [200,214]. If a similar link of the *Folr1*-folate interaction to cytoskeletal remodeling can be found in mammals, then a fresh interpretation of the folate-responsiveness of human and mouse NTD occurrences should be undertaken. Perhaps supplemental availability of folate to the *Folr1* receptor can maximize its activity, and can also compensate for weaknesses in other unrelated modules that also regulate the cytoskeleton.

### 8.4. Heterogeneity of Effects of Folate on Neural Tube Closure?

Overall, the mouse NTD mutants that interact with folate present a complex story that suggests that some folate effects on NTD risk may function through connections to the known metabolic roles of folate; other folate effects on NTD may be through previously unknown roles in ciliogenesis and regulation of the cytoskeleton. Given the great variety of cellular functions essential for successful neural tube closure, abundant levels of folic acid may be important via different aspects of its function in multiple aspects of the closure process, such as cell proliferation, regulation of a complex repertoire of developmental gene signaling via DNA and histone methylation, *Hedgehog* signaling, and cytoskeletal-driven bending of the neural plate. Given the genetic heterogeneity of NTD, individual cases may have different types of weakness in the closure process and interact with the aspect of supplemental folate that matches the functional deficit.

### 8.5. Mouse NTD Prevention by Formate

A new set of NTD mutants in folate-pathway genes is emerging. The genes are all involved in folate one-carbon metabolism in the mitochondria, the process that supplies one-carbon formate molecules to the cytosol for nucleotide biosynthesis and DNA methylation [6]. The reported mutants, which have between 25% and 100% exencephaly, are in genes *Amt*, *Gldc*, *Mthfd1l* and *Slc25a32* [170,215,216,217]. It appears that, whereas most of the non-mitochondrial folate-pathway mutants previously reported [201] do not have NTDs (the exception being *Folr1*), almost all of the mitochondrial folate-pathway mutants reported to date have exencephaly (with occasional craniorachischisis); none have spina bifida. The exception among the mitochondrial folate-pathway mutants that does not cause NTD is *Mthfd2*, which has redundancy with *Mthfd2l* [218].

Interestingly, in three of the mitochondrial mutants (*Gldc*, *Mthfd1l*, *Slc25a32*), the frequency of NTDs is reduced by between 60% and 100% by sodium formate supplementation (in drinking water) to the pregnant dams [170,216,217]; testing of the *Amt* mutant with formate has not been reported. In none of these mutants has folate/folic acid treatment reduced the NTD frequency.

A further example is the curly tail mouse strain, in which 10–20% of embryos have spina bifida. Folic acid has no preventative effect in curly tail strain embryos, but nucleotide precursors reduce spina bifida by 85% [219]. Recently, expression of the mitochondrial folate-pathway gene, *Mthfd1l*, was found to be 50% deficient in curly tail strain embryos and, as with the *Mthfd1l* null mutant (above), treatment of curly tail strain dams with prenatal sodium formate was found to reduce the spina bifida frequency by 75% [220].

Because the main source of one-carbon units for cytoplasmic processes comes from the mitochondria, any failure in a step of mitochondrial folate one-carbon metabolism results in a deficiency of one-carbon components (formate) for essential cellular processes, such as DNA synthesis and methylation. Momb et al. [216] suggest that a metabolic block in the mitochondria creates a “formyl trap”, starving the cytoplasm of formate, even in the presence of plentiful folate. It may be that formate, rather than folate, supplementation could be helpful in a similar category of human NTDs in which folate supplementation has no preventative effect.

## 9. An Overview of the Genetics of NTD

### 9.1. Genetic Architecture of Human NTD

Understanding of the genetic architecture of human NTD is largely derived from two approaches: family-based data and population-based data. The family-based approach led to the multifactorial model that explains the pattern of recurrence of NTD in families. Rates of recurrence of NTD in various types of relatives of NTD cases demonstrate that NTD have a strong genetic etiology, with a 30- to 50-fold increased rate of occurrence in siblings of NTD cases, compared to a population rate of about one per 1000 births [11,47]. The pattern of recurrence rates of NTD in other types of relatives does not fit simple single gene locus inheritance, but generally fits predictions for multiple gene locus inheritance, where the effects of variants at the multiple genes add together with each other and with environmental influences. We have summarized the foundational studies previously [12]. Conceptually, the genes involved may be unequal in effect [221,222] and the various genes contributing to risk of NTD in an individual case can be imagined to differ in impact, some having fairly large effects on risk, some having moderate effects, and some, perhaps many common polymorphisms, having small effects. A derivative of quantitative genetics, the multifactorial model assumes that individual cases, even with the same type of NTD, will have different subsets of the risk genes. 

Population-based studies aimed at finding statistical associations with single nucleotide polymorphisms (SNPs) in over 200 candidate genes have found a few weak associations often lacking statistical significance [1,223,224]. Association studies likely have been hampered by the heterogeneity of etiology of NTD at different locations along the neural tube, mismatch of candidate genes to the types of NTD in the pool of cases studied, dilution of power by use of pools of multiple types of NTD, and lack of adequate sample size for statistical significance of modest associations. 

Studies based on sequencing of coding regions of candidate genes usually are done as a population-based approach, often using pools of various types of NTD cases [10]. This approach does not depend on statistical association, only on the ability to identify sequence changes that affect gene function. As the multifactorial model for NTD predicts that individual cases have different combinations in subsets of the gene variants that cause risk of NTD, sequencing a pool of cases may detect few mutations at any given NTD risk gene sequenced. The pattern of findings fits this prediction. A few putative mutations have been found at each of some of the PCP system loci that have been sequenced in several pools of various types of NTD [10]. When examined, the mutations are usually inherited from a normal parent. The multifactorial concept predicts that the NTD cases have also inherited additional variants or mutations at other genes, probably from the other parent. Sequencing of coding regions of five PCP genes in a pool of 36 human craniorachischisis cases has yielded putative mutations at either of two genes, in about 13% of cases [181]. Similarly, exon sequencing of 191 candidate genes in a pool of 94 cases, most of which were anencephaly, has yielded 21 putative mutations affecting 19 genes [209] in about 22% of cases. In this study, consistent with multifactorial etiology, more of the additional rare/novel sequence variants that might have deleterious effects are found in each of the NTD cases than in the controls. The good yields from these two studies [181,209] perhaps demonstrate that increased effectiveness can be conferred by use of pools of only one type of NTD at a time. It should be noted that the usual sequencing approach cannot detect mutations in the more distant regulatory regions that direct the expression of genes to specific tissues (enhancers), and many such regulatory mutations at previously studied candidate genes may yet be discovered.

A current hypothesis about the genetic architecture of various multifactorial, post-reproductive diseases (“complex traits”) [225] suggests that a large proportion of the genome can have some impact on a “complex trait” disease. This concept is also a long-standing component of genetic selection theory based on empirical data for quantitative traits [226]. The genetic architecture likely differs between NTD and “complex traits” or quantitative traits because of the effects of genetic lethality of NTD on gene frequencies. For NTD, perhaps the important goal is to identify the variants of major effect whose combinations account for most of the risk of NTD, not all of the variants that can add smaller effects. There are probably many variants of major effect.

The common forms of NTD that are explained by the multifactorial model are non-syndromic, meaning the only primary defect is the defect in neural tube closure. 

There are some rare human syndromes of multiple unrelated defects that include anencephaly or spina bifida, and they are caused by chromosomal defects or Mendelian mutations [227]. They are not included in studies of common, non-syndromic NTD.

### 9.2. The Role of Environmental Effects

For a multifactorial trait, it is expected that environmental factors contribute to the trait to some degree. Theoretically, the effects of environmental factors add to the genetic risk factors in individuals. Individual embryos with a load of genetic risk factors but marginal ability to close the neural tube may be hampered enough by the additional effects of environmental factors to fail to close, whereas an exposed embryo with fewer genetic risk factors would still be able to successfully close. Folate supplementation (Section 8) is an environmental factor with a large effect that apparently compensates for the genetic risk factors in some way. Some other environmental factors are deleterious, with much weaker but statistically detectable effects. They include maternal diabetes, maternal obesity, maternal hyperthermia during pregnancy, parental occupations and exposure to chemicals, and a few pharmaceuticals [1].

### 9.3. The Role of Mouse Mutants in NTD Genetics

The almost 300 mouse NTD mutants cannot model the human genetic architecture of NTD. The mouse mutants are maintained on inbred strain backgrounds, within which there is essentially no genetic variation between individuals. Very few strain backgrounds are used, and the strains themselves are a very limited sample of the variation in the mouse species’ genome. Some mutations show different penetrance or expressivity of NTD on different strain backgrounds, demonstrating their potential for multifactorial genetics. In the studies of digenic effects, to test the combined effects of two different genes on NTD risk, the specific digenic combination of interest is created by crosses between mutant stocks. Unexpected genetic interactions are unlikely to be discovered in this hypothesis-driven system.

The mouse mutations are usually highly penetrant recessive “knockouts” that cause loss of function of the gene, and for many of the genes, these cause syndromes of multiple defects. In general, gene loci that cause syndromic phenotypes from null (knockout) mutations may present non-syndromic phenotypes from hypomorphic (reduced function) mutations. An example in mice is the kidney defect and cleft lip syndrome caused by a null mutation at *Wnt9b* versus the non-syndromic cleft lip phenotype from a hypomorphic mutation at *Wnt9b* [228]. Therefore, the mouse mutants serve to identify candidate loci for human NTD despite the severity of the null mutant phenotypes. 

The use of candidate genes from mice should be tempered by awareness of the differences in the morphological process between human and mouse neural tube development (Section 4.1), and ideally should be informed by gene expression data from other species whose neural tube closure is morphologically more similar to human, such as pig and rabbit.

Many of the candidate genes that have been tested in human NTD cases are based on various hypotheses, such as roles related to folate metabolism or folate transport, and the lack of finding gene variants associated with human NTD concurs with the mouse mutants; i.e., they do not cause NTD in mouse mutants. 

The pools of human cases studied usually focus on spina bifida. The majority of the NTD genes in mouse mutants cause cranial NTD, and based on the regional heterogeneity of etiology discussed in Section 3 and Section 5, these genes would not be expected to participate in human spina bifida. This mismatch may contribute to the disappointing results from some studies.

Two successful sequencing studies are discussed in Section 9.1. Both demonstrate the value of mouse NTD mutants as candidate genes. The study of a few of the PCP genes in craniorachischisis cases [181], based on the observation that nearly all genetic craniorachischisis in mice is caused by PCP gene mutations [12] has been fruitful, yielding putative mutations from 13% of cases. In a similar study [209] based on mostly anencephalic cases, about half of the 19 genes with putative mutations have mouse NTD mutants at the same gene or a member of the same gene family.

## 10. Concluding Remarks

Various insights emerge from an overview of the many studies of neural tube closure. The mammalian way of forming a neural tube by elevating and bending the lateral edges of a neural plate to meet in the midline is an ancient mechanism that evolved before vertebrates. Several systems contribute to forming the neural tube. Among them are the planar cell polarity/convergent extension system; the medial hinge point and related cell cycle phenomena; the dorsolateral hinge point and the *Sonic hedgehog* cilia system on which it depends; the actomyosin and cytoskeletal system responsible for some bending of the neuroepithelium; the various types of cell projections and the *Ephrin* system that facilitate contact and fusion. Each of these systems involves many genes. Despite the understanding of many details of the genetic determination of neural tube development, the genetic causes of human NTD are mostly undiscovered. 

The evidence that the planar cell polarity system is involved in various aspects of neural tube closure in addition to convergent extension, such as the cytoskeleton, ciliogenesis, and the actomyosin system, offers an explanation for the observed role of planar cell polarity genes in exencephaly/anencephaly and spina bifida as well as in craniorachischisis.

Although the mouse has been used extensively as the mammalian model for human NTD, the development of the neural tube in other mammals, such as pig and rabbit, is more similar to human in morphology. Gene expression arrays of regions of neural folds from these species during elevation and closure might point to the subset of genes and mechanisms previously identified in mice that are likely to be involved in human neural tube closure. 

The vertebrate head evolved as an “add-on” to the pre-vertebrate body plan and its neural tube. The formation of the cranial neural tube consequently differs in several ways from that of the spinal region. For example, the mouse cranial mesoderm has a much greater role in neural tube closure and etiology of NTD. The neuroepithelial actomyosin system is also more important in the cranial region than in the spinal region. The large number of genes involved in these two systems may account for the predominance of genes causing exencephaly among mouse NTD mutants. The genes identified by these mutants may be good candidates for examination in the etiology of human anencephaly. 

Cranial NTD occur more frequently in females than in males. This phenomenon is seen in humans and in nearly all mouse NTD mutants examined. Recent advances in the understanding of epigenetics suggest the hypothesis that the second, inactive X chromosome of female embryos is a “heterochromatin sink” that sequesters essential epigenetic factors, altering expression of genes in the rest of the genome and thereby increasing the risk of NTD. The limitation of the effect to cranial NTD suggests that specific genes are involved. 

It is well established that peri-conceptional dietary folic acid supplementation reduces the risk of occurrence of NTD in humans and in some mouse mutants, but the genetic risk of folate-responsive NTD does not seem to involve genes in the folate metabolic pathway in either species. There is recent evidence for a function of folate-pathway genes that is separate from the established metabolic functions in DNA methylation and nucleotide synthesis. In Xenopus, the *Folr1* membrane protein that takes folate into cells seems to act also as a regulator of the cytoskeleton and of cell adhesion remodeling in the neural plate. Secondly, independent evidence in Xenopus demonstrates a requirement for folate in ciliogenesis, which is known to be involved in NTD. These observations may shift the focus of candidate gene research in folate-responsive human NTD away from the metabolic functions of folate.

There are many examples in mouse mutants of NTD caused by combinations of mutant genes at two or more loci from the same or different genetic pathways. Usually, the molecular mechanism of interaction between the mutant genes is unknown. The mechanism of the interaction effect can be surprising, as in the case of the *Msx2/Dlx5* double mutant, where the combined effect of the two mutant genes alters the balance of splice forms of a third gene, *EphA7*. The extensive study of the planar cell polarity system has generated several examples of digenic combinations of different planar cell polarity genes that cause a variety of types of NTD. The observations in mice suggest that many of the cases of human NTD in which a putative mutation has been detected at one planar cell polarity gene locus may well harbor an undetected mutation at a second planar cell polarity gene locus or other type of gene.

Overall, the cumulative data show that whereas the neural tube appears to be a single anterior–posterior structure, in contradiction, the gene expression, cell behavior, and closure systems occupy a variety of different regions. The neural tube along its length is neither homogeneous nor segmented. Each gene and mechanism is expressed in areas along the length that are different from the expression patterns for many of the other genes or mechanisms so that each linear area is the result of a unique combination. NTD may be a series of genetically distinct defects occurring along a contiguous physical structure. Setting aside the planar cell polarity system mutations, the remainder of human NTD may have genetic etiology that varies by anterior–posterior location of the lesion, a concept that could inform human genetic studies in search of causative variants. For example, families within which there are both anencephalic and spina bifida cases may tend to have mutations in the DLHP system. Perhaps the most important conclusion from this review is recognition of the degree to which the neural tube is regionalized along the anterior–posterior axis.

## Figures and Tables

**Table 1:** Ways in which the neural tube can fail to form.

Lack of convergent extension
Lack of medial hinge point (MHP) ^1^
Lack of dorsolateral hinge point (DLHP) ^1^
Lack of neuroepithelial bending by apical constriction (cranial region)
Lack of structural integrity of the neuroepithelium
Lack of support from surrounding mesenchyme Lack of midline fusion of neuroepithelium and/or surface ectoderm Interference by excessive anterior-posterior curvature of the neural plate

^1^ Shown in Figure 2.

**Table 2:** Regional differences in mechanisms for neural tube closure.

Mechanism	Difference between Regions of Neural Tube
Convergent extension	Absent in forebrain
Notochord in head	Absent in forebrain
Notochord in spinal region	Absent at posterior neuropore during closure
Level of *Shh* expression	Gradient; lowest in posterior neuropore
Medial Hinge Point	Absent in posterior neuropore
Dorsolateral Hinge Point	Absent in upper spinal region
Convex mesenchymal expansion	Midbrain only
*Grhl2*, *Grhl3* expression	Cranial, caudal and upper spine differ
*Ephrins* and Ephrin receptors	Different members of gene family regionally
*Hox* gene expression	Absent in midbrain and forebrain regions
Closure initiation site spacing	Caudal closure furthest from an initiation site
“Zipping” vs. simultaneous closure	Regional differences in axial bending
Meeting of neural folds in midline	Contact of neuroepithelium vs. surface ectoderm
Ruffles versus filopodia	Forebrain, midbrain, hindbrain, spine differ
Apical actomyosin contractility	Required for cranial closure, not spinal
Neural crest emigration	Spinal after closure; cranial before closure
Apoptosis	Required for cranial closure, not caudal
